# Supplementary material for: Does problem-based learning improve patient empowerment and cardiac risk factors in patients with coronary heart disease in a Swedish primary care setting? A long-term prospective, randomised, parallel single randomised trial (COR-PRIM)
Source: BMJ Open. 2023 Feb 24;13(2):e065230. doi: 10.1136/bmjopen-2022-065230 (PMC9972427; doi:10.1136/bmjopen-2022-065230)
Supplement: Supplementary data [file bmjopen-2022-065230supp001.pdf]

Supplemental Material 1

Linköpings universitet
HÄLSOUNIVERSITETET
COR-PRIM study
Department of social – and welfare studies
Linköping University, Campus Norrköping
2013. Anita Kärner phone: 011–363506

## Responding letter regarding participation in the COR-PRIM study

**From: Patients name………………………………………………………** (Written before sending out)

For about a week ago you received a written patient information about the COR-PRIM study, a test of a new treatment with pedagogical elements in primary care. Thereafter you were contacted by Anita Kärner, university lecturer and responsible for the study, and responded orally yes/wanted to consider more about the enquiry of participating in the study.
For you to be able to participate a written answer is needed as well. Put an X in a suitable box below. We want to emphasize that participation is totally voluntarily, and you can without motivation abstain from answering this request.

☐ I want to participate

☐ I do not want to participate. Reason (voluntarily information).

☐ I wish to be contacted again for more information before I decide. This box can also be filled in for you who want to participate. You can write question on the backside of this response letter

Date:……………………………………………………..

Signature:…………………………………………………………………………………………

For you who want to be contacted again. Write telephone number and times when you can be contacted again on the backside of this letter.

Anita Kärner
University lecturer
Department of social – and welfare studies
Campus Norrkoping
Linköping University
Anita.Karner@liu.se
Phone: 011–363506

Staffan Nilsson
General practitioner
Primary Care Vikbolandet
County council Östergötland
Staffan.Nilsson@lio.se
Phone: 010–1030000
