# Supplementary material for: Does problem-based learning improve patient empowerment and cardiac risk factors in patients with coronary heart disease in a Swedish primary care setting? A long-term prospective, randomised, parallel single randomised trial (COR-PRIM)
Source: BMJ Open. 2023 Feb 24;13(2):e065230. doi: 10.1136/bmjopen-2022-065230 (PMC9972427; doi:10.1136/bmjopen-2022-065230)
Supplement: Supplementary data [file bmjopen-2022-065230supp002.pdf]

Supplemental Material 2 *BMJ open*

Title of the article:
Does problem-based learning improve patient empowerment and cardiac risk factors in patients with coronary heart disease in a Swedish primary care setting? A long-term prospective, randomized, parallel single randomized trial (COR-PRIM).

Authors: Christina Andreae, Pia Tingström, Staffan Nilsson, Tiny Jaarsma, Nadine Karlsson and Anita Kärner Köhler.

Supplement 2. Description of the PBL intervention

The PBL intervention consisted of group learning sessions in primary care led by district nurses who were advanced trained in PBL for two days by the research group. Two of the authors (AKK and PT) supported and tutored the district nurses every month in their development as PBL tutors. Tutoring groups consisted of 6-9 patients and participated in 13 learning sessions (2 hours) over one year. The sessions were every week for the first month, the two next months included two sessions/month, and the next five sessions were at 16, 20, 26, 39, 52 weeks after the start.

A problem-solving process (a PBL-model) supported the patient's formulation of mutual questions and learning goals and individual self-care goals, which they wrote and reflected on in an individual study guide. Triggers e.g., texts including real life scenarios or images were used to start the learning process about CHD, symptoms such as: chest pain, shortness of breath, swollen legs and signs of high blood pressure, cholesterol levels, lifestyle changes, in diet, physical activity, medicines, mental outcomes e.g., depressive mood, anxiety and fears, job life and stress, sex life. Importantly, the patients chose in which order the different scenarios were chosen to work with. This was a way to empower them and make the learning relevant to their situation. The district nurses supported and challenged the patients to select evidence-based literature. When the patients could not solve the questions, resource persons e.g., a general practitioner, physiotherapist were invited. These meetings were in between the group meetings and interested relatives were welcome and did attend.

Patients randomized to the control group received with the same interval as the PBL group, home-sent evidence-based patient information about self-care reflecting a cognitive intervention but did not participate in critical or reflective learning. Group effects were assessed after 11 sessions over one year.
