# Supplementary material for: Does problem-based learning improve patient empowerment and cardiac risk factors in patients with coronary heart disease in a Swedish primary care setting? A long-term prospective, randomised, parallel single randomised trial (COR-PRIM)
Source: BMJ Open. 2023 Feb 24;13(2):e065230. doi: 10.1136/bmjopen-2022-065230 (PMC9972427; doi:10.1136/bmjopen-2022-065230)
Supplement: Author's manuscript [file bmjopen-2022-065230.draft_revisions.pdf]

BMJ Open

BMJ Open is committed to open peer review. As part of this commitment we make the peer review history of every article we publish publicly available.

When an article is published we post the peer reviewers' comments and the authors' responses online. We also post the versions of the paper that were used during peer review. These are the versions that the peer review comments apply to.

The versions of the paper that follow are the versions that were submitted during the peer review process. They are not the versions of record or the final published versions. They should not be cited or distributed as the published version of this manuscript.

BMJ Open is an open access journal and the full, final, typeset and author-corrected version of record of the manuscript is available on our site with no access controls, subscription charges or pay-per-view fees (http://bmjopen.bmj.com).

If you have any questions on BMJ Open's open peer review process please email info.bmjopen@bmj.com

BMJ Open

Long-term effects of problem-based learning on self-management and risk-factors in coronary heart disease - A randomized study in primary care (COR-PRIM)

Journal:	*BMJ Open*
Manuscript ID	bmjopen-2022-065230
Article Type:	Original research
Date Submitted by the Author:	01-Jun-2022

Complete List of Authors:	Andreae, Christina; Linkoping University Department of Health Medicine and Caring Sciences,<br>Tingström, Pia; Linkoping University Department of Health Medicine and Caring Sciences<br>Nilsson, Staffan; Linkoping University Department of Health Medicine and Caring Sciences<br>Jaarsma, Tiny; Linkoping University Department of Health Medicine and Caring Sciences<br>Karlsson, Nadine; Linkoping University Department of Health Medicine and Caring Sciences<br>Köhler, Anita Kärner; Linkoping University Department of Health Medicine and Caring Sciences

Keywords:	Coronary heart disease < CARDIOLOGY, PRIMARY CARE, EDUCATION & TRAINING (see Medical Education & Training)

SCHOLARONE™
Manuscripts

I, the Submitting Author has the right to grant and does grant on behalf of all authors of the Work (as defined in the below author licence), an exclusive licence and/or a non-exclusive licence for contributions from authors who are: i) UK Crown employees; ii) where BMJ has agreed a CC-BY licence shall apply, and/or iii) in accordance with the terms applicable for US Federal Government officers or employees acting as part of their official duties; on a worldwide, perpetual, irrevocable, royalty-free basis to BMJ Publishing Group Ltd ("BMJ") its licensees and where the relevant Journal is co-owned by BMJ to the co-owners of the Journal, to publish the Work in this journal and any other BMJ products and to exploit all rights, as set out in our licence.

The Submitting Author accepts and understands that any supply made under these terms is made by BMJ to the Submitting Author unless you are acting as an employee on behalf of your employer or a postgraduate student of an affiliated institution which is paying any applicable article publishing charge ("APC") for Open Access articles. Where the Submitting Author wishes to make the Work available on an Open Access basis (and intends to pay the relevant APC), the terms of reuse of such Open Access shall be governed by a Creative Commons licence – details of these licences and which Creative Commons licence will apply to this Work are set out in our licence referred to above.

Other than as permitted in any relevant BMJ Author's Self Archiving Policies, I confirm this Work has not been accepted for publication elsewhere, is not being considered for publication elsewhere and does not duplicate material already published. I confirm all authors consent to publication of this Work and authorise the granting of this licence.

For peer review only

Long-term effects of problem-based learning on self-management and risk-factors in coronary heart disease - A randomized study in primary care (COR-PRIM)

Christina Andreae[1,2], Pia Tingström[1], Staffan Nilsson[3], Tiny Jaarsma[1,4] Nadine Karlsson[1] and Anita Kärner Köhler[1]

1. Department of Health, Medicine and Caring Sciences, Linköping University, Linköping, Sweden.

2. Centre for Clinical Research Sörmland, Uppsala University, Eskilstuna, Sweden.

3. Primary Health Care Center Vikbolandet, and Department of Health, Medicine and Caring Sciences, Linköping University, Linköping, Sweden.

4. Julius Center, University Medical Center Utrecht, Utrecht, the Netherlands.

Corresponding author: Christina Andreae, PhD, RN, Department of Medicine, Region Sörmland, 63188 Eskilstuna, Sweden

Email: christina.andreae@liu.se

Tel: +4616 10 44 07

For peer review only

Abstract

Objectives: To investigate long-term effects of a one-year problem-based learning (PBL) on self-management and cardiac risk factors in patients with coronary heart disease.

Design: A prospective, randomized, parallel single centre trial.

Settings: Primary care settings in Sweden

Participants: 157 patients with stable coronary heart disease (CHD) completed the study. Subjects with reading and writing impairments, mental illness or expected survival less than one year were excluded.

Intervention: Participants were randomized and assigned to receive either PBL (intervention) or home-sent patient information (control group). In this study participants were followed-up at baseline 1, 3 and 5 years.

Primary and secondary outcomes: Primary outcome was patient empowerment (SWE-CES) and secondary outcomes general self-efficacy (GSES), self-rated health status (EQ-VAS), high-density lipoprotein (HDL) cholesterol, Body Mass Index (BMI), weight and smoking. Outcomes were adjusted for sociodemographic factors.

Results: The PBL intervention group resulted in a significant improved change in SWE-CES over the five-year period (Mean [M], 39.39; 95% confidence interval [CI], 37.88-40.89) compared to the baseline (M, 36.54; CI, 35.40-37.66). PBL intervention group increased HDL level (M, 1,39; CI, 1.28-1.50) compared to baseline (M, 1,24; CI, 1.15-1.33) and for EQ-VAS (M, 77.33; CI, 73.21-81.45) compared to baseline (M, 68.13; CI, 63.66-72.59) while these outcomes remained unchanged in the control group. There were no significant differences in BMI, weight, or scores on GSES, neither between nor within groups over time. The overall proportion of smokers was significantly higher in the control group than in the experimental group. Covariates age, sex, education, and marital status emerge both as healthy and cardiac risk factors.

For peer review only - http://bmjopen.bmj.com/site/about/guidelines.xhtml

For peer review only

Conclusion: PBL intervention in primary care had positive long-term effects on self-management and cardiac risk factors in patients with CHD. The results indicate that sex, age, and sociodemographic factors should be considered when recruiting patients in need of empowering self-management strategies after CHD.

Keywords: Coronary disease, empowerment, heart disease risk factors, long-term, primary health care, problem-based learning, randomized controlled trial.

For peer review only

Trial registration: The study was registered at ClinicalTrial.gov with number NCT01462799.

Strengths and limitations of this study:

- The PBL intervention was provided for 1 year and patients were followed for 5 years, which provides a deep understanding of behavioral changes in patients with CHD.
- One-year PBL programme may strengthen patient empowerment and health status as well as reducing cardiac risk factors but may not result in adherence to guidelines.
- Most of the patients were retired and co-habiting, which may imply that lifestyles were influenced by partners.
- PBL education aiming to improve patient empowerment in cardiac rehabilitation may be possible to apply in other primary care settings. Further experimental studies would have to consider sociodemographic factors to improve self-management and cardiac risk factors in CHD.
- Patients could choose to participate, but few chose to participate in group education, a web-based PBL education may therefore be a future option.

For peer review only - http://bmjopen.bmj.com/site/about/guidelines.xhtml

Introduction

Nearly 126 million people globally suffer from coronary heart disease (CHD), the leading cause of death worldwide [1,2]. Important treatment goals include slowing down the underlying atherosclerosis process by targeting hypertension, high cholesterol, diabetes, overweight, tobacco use, alcohol consumption, physical inactivity, and poor diet [3]. Cardiac rehabilitation consists of multidisciplinary interventions where patients are offered tailored education and counselling with the goals of improving health behavior for sustainable secondary prevention [3]. Several studies have investigated the effect of interventions such as nurse telephone follow-up or group education, and described the effects on illness perception, self-efficacy, behaviour change and cardiac risk factors [4-6]. The effects of several programmes on cardiac risk factors or behaviour were found from 3 months up to 2 years.

Patients' attendance of cardiac rehabilitation appears to be low, and risk factors remain or even deteriorate from the first to second cardiac event [7-9]. For example, persistent smoking is common after discharge from hospital, and intentions to quit smoking in the near future remain low. This regards also overweight and physical activity after a cardiac event, many patients do not lose weight and nearly half of patients perform no physical activity and do not make changes to their physical status [8]. Thus, primary care has great challenges motivating patients to achieve healthy lifestyles.

Empowering patients to take control over the disease, and making them aware of factors that affect illness, have positively impacted on health outcomes in chronic diseases [10]. Problem-based learning (PBL) is a method that empowers patients to become aware of how to reduce risk factors in chronic diseases [11]. PBL is a cognitive educational model that promotes self-learning where critical and reflective thinking are important components of learning outcomes and enhanced self-management. PBL is process-oriented, meaning that learning skills are developed through active, creative, and cognitive processes. Previous knowledge is placed in

For peer review only - http://bmjopen.bmj.com/site/about/guidelines.xhtml

the light of real situations where knowledge is developed in a context together with others, all led by a trained PBL tutor [12]. A focus group intervention with problem solving intervention in a cardiac population showed that an eight-week focus group sessions resulted in significant improvements of stress management, dietary intake and physical activity [13]. The first results from a one-year follow-up in a coronary heart disease in primary care study (COR-PRIM) showed no differences in patient empowerment or self-efficacy between PBL intervention and patients who received home-sent patient information (controls). However, significant differences in secondary outcomes – i.e., body mass index, body weight and high-density lipoprotein cholesterol (HDL-C) – were found between the groups, with the PBL group performing favourably [14].

Although cardiac rehabilitation in Sweden has recently been assessed to be high quality [15] participation remains low. Only a few interventions incorporating a holistic view of health compromising psychological- and social aspects of health behaviors have been performed on the group level. PBL may empower self-management. Long term follow-up of the effects of PBL in CHD on patient empowerment, self-efficacy, health status and cardiac risk factors assessed in primary care settings has not previously been reported. The purpose of this study was to investigate the long-term effects on patient empowerment, self-efficacy, health status and cardiac risk factors in patients with CHD of a one-year PBL intervention in primary care, compared to home-sent patient information.

Methods

Design, procedure, and randomization

The COR-PRIM study was a prospective, randomised, parallel, and single centre trial designed to investigate whether one year of a PBL programme had long-term effects on

For peer review only - http://bmjopen.bmj.com/site/about/guidelines.xhtml

patient empowerment, self-efficacy and cardiac risk factors [12]. The study protocol is registered at ClinicalTrial.gov, with the registration number NCT01462799. Patients diagnosed with CHD verified by percutaneous coronary intervention (PCI) or coronary artery by-pass surgery (CABG) and CABG+PCI or myocardial infarction within 6-12 months prior to the intervention and who had previously completed cardiac rehabilitation were eligible for the study. Additional criteria were that patients were stable in their heart disease, pharmacologically optimized in the last month before inclusion and, if applicable had completed cardiac school in the clinic. Patients with impaired ability to communicate and read in Swedish, verified psychiatric illness or short expected survival (of less than one year) were excluded [12]. The recruitment started in November 2011 and assessments of primary and secondary outcomes were collected until November 2019. Nurses at outpatient cardiac clinics identified eligible patients through medical records. These patients were then invited to participate in the study at a regular nurse-led cardiac follow-up visit. Patients received oral and written study information from a research assistant, and written consent was collected from those interested in participating.

In COR-PRIM, a total of 157 patients were randomized and assigned to either problem-based learning intervention (PBL) or home-sent patient information (control group). The randomization procedure was based on block randomization with 18 study numbers based on a minimum of 12-18 study participants. Block randomization was performed using sealed and unmarked envelopes. An assistant, blinded to the randomization and located outside the research setting, randomly allocated patients to either the PBL group (experimental) or home sent patient information (control group) at a 1:1 ratio [12]. Nurses who performed the PBL intervention were not blinded, as the intervention was based on problem-based learning, which is different from regular care. The PBL intervention consisted of group learning

For peer review only - http://bmjopen.bmj.com/site/about/guidelines.xhtml

sessions in primary care led by nurses who had advanced training in leading problem-based learning. Tutoring groups consisted of 6-9 participants and participated in 13 sessions over one year. Patients randomized to the control group received home-sent patient information reflecting a cognitive intervention, but did not participate in critical or reflective learning. Group effects were assessed after 11 sessions over one year [12].

Patient and public involvement

There were no patient or public involvement in the study.

Sample

Of the initial 446 invited participants, 289 were excluded. Of the latter, 23 did not fulfil the inclusion criteria, 246 declined to participate, and 20 did not respond to the invitation. The final study group consisted of 157 participants, of whom 79 were assigned to the PBL group and 78 assigned to the control group. At the three-year follow-up there were 5 dropouts, 2 participants (1 from the control group and 1 from the PBL group) had not completed the study for personal reasons, and 3 participants had died (2 from the control group and 1 from the PBL group), leaving n=77 participants (PBL) and n=75 (control group) at the three-year follow up. At the five-year follow-up there were 9 dropouts, 2 participants (1 from the control group and 1 from the PBL group) had advanced in their illness, and 7 participants had died (3 from the control group and 4 from the PBL group). Analyses conducted for the five-year follow-up period was n=72 (PBL) and n=71 (control group) (Figure 1). Sample size was calculated based on the primary outcome variable i.e., patient empowerment. The expected means for the Swedish Coronary Empowerment Scale (SWE-CES) were 30 and 36 for the control group and the PBL group respectively. With an estimated significance level of α=5% and a power of 1-β=80%, the sample size resulted in a minimum of 63 participants in each group [12].

For peer review only - http://bmjopen.bmj.com/site/about/guidelines.xhtml

Primary and secondary outcome measures

Patient empowerment was the study's primary outcome and was assessed at baseline, 1, 3 and 5 years (i.e., baseline, time 1, time 3 and time 5 respectively). Secondary outcomes included general self-efficacy, health status, high-density lipoprotein cholesterol, body mass index, weight and smoking, and were assessed at baseline, 1, 3 and 5 years.

Measurement

Sociodemographic variables and covariates

Sociodemographic variables consisting of age, sex, education level, marital status, place of residence, employment and smoking were determined by self-reported questionnaires. The covariates consisted of age, sex, education level and marital status.

Self-reported measures

The Swedish coronary empowerment scale (SWE-CES) measures how patients achieve goals, overcome barriers for goals achievement, and use the strategies necessary to make self-care choices. The strategies include dimensions of coping in managing disease, stress and dissatisfaction and also readiness to make health changes. The instrument contains 10 items that have 5 response alternatives ranged by Likert type options from 1 to 5. The total score ranges between 10 and 50, where high scores imply high level of patient empowerment. Four subscales measure different aspects of patient empowerment [16]. In this study, the total scores of the SWE-CES scale were used. Internal consistency reliability indicated acceptable values with Cronbach's alfa 0.751 [17].

Self-efficacy was measured by using the general self-efficacy scale (GSES). The instrument consists of 10 items that measure a person's belief in their own ability to implement behavioral changes in order to reduce risk factors for unhealthy lifestyles. Items are rated on a four-point Likert type scale ranging from 1 (not at all true) to 4 (exactly true). The total score

For peer review only - http://bmjopen.bmj.com/site/about/guidelines.xhtml

For peer review only

ranges from 10 to 40, where a high total score indicates higher general self-efficacy [18]. Self-efficacy has been used to evaluate interventions to strengthen the self-care capacity of patients with various chronic diseases such as diabetes [19,20] and patients with CHD [21-23]. It has been translated and psychometrically evaluated among a general Swedish population [24]. In this study, internal consistency reliability indicated acceptable values with Cronbach's alfa 0.914.

Health status

Self-rated health was assessed using EuroQol (EQ-VAS). The scale represents patients' overall health status and consists of a score between 0 and 100, where 0 indicates worst health and 100 the best health [25]. EQ-VAS has shown acceptable construct validity across populations [26].

Cardiac risk factors

High density lipoprotein cholesterol (HDL-C) was assessed using blood samples collected and analysed according to normal clinical routines. Anthropometric measurements of body size included length, weight, and Body Mass Index (BMI). Length and weight were measured in light cloths and with shoes removed. BMI is a widely used clinical measure and is recommended as indicator for defining obesity in adults. It is, furthermore, a reliable anthropometric measure in predicting metabolic syndromes [1,27]. BMI was calculated by dividing weight by the square of height in meters using the formula (weight (kg) / height (m^2)) [28]. Tobacco use was self-reported at each measure point.

Statistical analysis

The descriptive statistics included mean standard deviation (SD) or frequencies percent (%). Independent sample t-tests or Chi-square tests were used to analyses differences in sociodemographic and data characteristics between groups. Because the occasions (baseline to five-year) are nested within individuals, we employed a two-level mixed linear model with

For peer review only - http://bmjopen.bmj.com/site/about/guidelines.xhtml

occasions ("time" at level 1) that are nested within individuals (level 2) to analyze continuous outcomes. The random part of the variance components model is individuals. The normality of continuous study outcomes was assessed using Shapiro-Wilk test. If the outcomes were non-normally distributed, they were log transformed prior to the analysis. Smoking is a binary outcomes and was analysed with a logistic mixed model. The statistical analysis of each study outcome was performed using a mixed linear model with treatment group, age and sex as fixed variables, with occasions nested within individuals, and an interaction of time by treatment group. The interaction (combined effect) between treatment group and time was tested using the likelihood ratio test. In the presence of an interaction, the analysis was stratified by treatment group, and a mixed model with occasions nested within individuals was performed separately for each group. These stratified analyses were adjusted in two models. Model 1 was adjusted for age and sex, and Model 2 was, furthermore, adjusted for educational level and marital status. The level of significance used was $p<0.05$. When the interaction term was not significant at 5% but was significant at 10%, a sensitivity analysis was performed by exploring the results of the analysis stratified by intervention group (PBL and control). The statistical analysis was performed in SPSS 27 and Stata 16.0.

Results

Sample

Of the total sample of 157 participants, the mean age was 68.7 years ± 8.5 and the majority were men n = 122, 77.7 % (Table 1). Almost half of the participants lived in urban areas, and the remaining lived small towns or the countryside. In total, 113 participants were retired and the remaining were in employment. Almost half of the participants had suffered from myocardial infarction, which occurred at a mean of 284 days before the start of the study.

For peer review only - http://bmjopen.bmj.com/site/about/guidelines.xhtml

Table 1. Baseline characteristics of the participants in the COR-PRIM study randomized to problem-based learning intervention (PBL) or home-sent patient information (control group).

	Total sample (N=157)	PBL (n=79)	Control (n=78)	p-value
Age year, mean (SD)	68.7 (8.5)	68.5 (9.2)	68.9 (7.7)	0.78[a]
Sex, n (%)				
Male	122 (77.7)	60 (75.9)	62 (79.5)	
Female	35 (22.3)	19 (24.1)	16 (20.5)	0.70[b]
Marital status, n (%)				
Cohabiting	115 (74.2)	60 (75.9)	55 (72.4)	
Living alone	40 (25.8)	19 (24.1)	21 (27.6)	0.71[b]
Education level, n (%)				
Compulsory	84 (54.2)	46 (58.2)	38 (50.0)	
Upper secondary	31 (20.0)	16 (20.3)	25 (19.7)	
University	38 (24.5)	17 (21.5)	21 (27.6)	0.43[b]
SWE-CES total score, mean (SD)	36.8 (5.3)	36.5 (4.9)	37.1 (5.8)	0.49[a]
GSES total score, mean (SD)	31.30 (5.13)	31.20 (5.44)	31.41 (4.82)	0.80[a]
EQ-VAS, mean (SD)	70.21 (17.9)	68.13 (19.5)	72.44 (15.80)	0.05 [a]
HDL-C, mmol/L, mean (SD)	1.27 (0.44)	1.24 (0.39)	1.30 (0.48)	0.42[a]
BMI, kg/m2, mean (SD)	27.10 (4.39)	27.12 (3.91)	27.07 (4.85)	0.95[a]
Smoking, n (%)	19 (12.1)	9 (11.4)	10 (12.8)	0.81[b]

For peer review only - http://bmjopen.bmj.com/site/about/guidelines.xhtml

SWE-CES=Patient empowerment, high score implies stronger empowerment;

GSES=General Self-Efficacy, high score implies high general self-efficacy.

EQ-VAS=health status; HDL-C=high-density lipoprotein cholesterol; BMI=Body Mass Index kg/m2;

a=Independent sample *t-test*

b=Chi-square test

For peer review only

At baseline, the majority had no symptoms of angina pectoris. Less than half of the participants had hyperlipidemia. Additional characteristics are available from a previous study [14].

The distribution for continuous outcomes for the PBL and control groups at the one, three and five-year follow-ups is illustrated with a summary of data including minimum, first quartile, median, third quartile and maximum in boxplots (Figure 2).

The results of the statistical analysis of study outcomes over time and by intervention are summarized below (see table 2). Results of the stratified analysis adjusted for age and sex only gave results similar to the model also adjusted for educational level and marital status. Therefore, table 2 presents only results adjusted for age, sex, educational level, and marital status.

For peer review only - http://bmjopen.bmj.com/site/about/guidelines.xhtml

Table 2. Differences in self-management and cardiac factors between problem-based learning intervention (PBL) vs home sent information (control group). Stratified analysis is adjusted for age, sex, educational level and marital status. Mixed linear model analyses were performed for (SWE-CES, GSES, EQ-VAS, HDL-C, BMI and Weight) and mixed logistic regression for (smoking).

	Outcome	*p*-value	N	PBL group Mean (SD) or n (%)	*p*-value	N	Control group Mean (SD) or n (%)	*p*-value
Overall effect of group	SWE-CES	0.558						
Time x group		0.086[b]						
Baseline		Ref	76	36.54 (4.9)	Ref	72	37.15 (5.8)	Ref
Time 1		0.386	57	37.79 (5.2)	0.119	54	36.57 (5.5)	0.720
Time 3		0.060	56	38.34 (5.7)	0.023	55	37.80 (5.7)	0.834
Time 5		0.025	49	39.39 (5.2)	< 0.001	47	37.21 (5.0)	0.721
Overall effect of group	GSES	0.791						
Time x group		0.331[c]						
Baseline		Ref	75	31.20 (5.44)	-	71	31.41 (4.82)	-
Time 1		0.201	61	31.07 (4.66)	-	58	31.88 (4.99)	-
Time 3		0.793	57	31.56 (5.26)	-	54	31.67 (5.56)	-
Time 5		0.123	55	32.69 (5.46)	-	52	31.83 (4.70)	-
Overall effect of group	EQ-VAS	0.875						
Time x group		0.022[a]						
Baseline		Ref	76	68.13 (19.53)	Ref	71	72.44 (15.80)	Ref
Time 1		0.038	59	74.64 (18.05)	0.026	60	75.30 (16.36)	0.501
Time 3		0.256	56	78.27 (14.84)	0.007	56	71.89 (18.33)	0.195
Time 5		0.462	57	77.33 (15.52)	0.031	51	72.06 (17.19)	0.137
Overall effect of group	HDL-C	0.555						
Time x group		0.016[a]						
Baseline		Ref	74	1.24 (0.39)	Ref	75	1.30 (0.48)	Ref
Time 1		0.075	59	1.37 (0.43)	0.003	60	1.23 (0.36)	0.781
Time 3		< 0.001	47	1.42 (0.55)	< 0.001	44	1.27 (0.37)	0.655
Time 5		0.036	58	1.39 (0.41)	< 0.001	55	1.27 (0.42)	0.459
Overall effect of group	BMI	0.962						
Time x group		0.061[b]						
Baseline		Ref	65	27.12 (3.91)	Ref	67	27.07 (4.85)	Ref
Time 1		0.951	58	26.83 (3.93)	0.076	56	27.83 (4.97)	0.100
Time 3		0.673	43	27.33 (4.22)	0.440	44	28.04 (4.77)	0.874

For peer review only - http://bmjopen.bmj.com/site/about/guidelines.xhtml

Time 5		0.897	53	27.11 (4.43)	0.701	52	27.78 (5.07)	0.862
Overall effect of group	Weight	0.772						
Time x group		0.047[a]						
Baseline		Ref	74	81.75 (13.61)	Ref	74	81.44 (17.45)	Ref
Time 1		0.739	61	81.77 (13.58)	0.085	61	84.05 (17.19)	0.045
Time 3		0.607	47	83.83 (15.52)	0.788	46	86.40 (18.64)	0.372
Time 5		0.426	56	84.52 (15.16)	0.864	57	86.15 (18.20)	0.408
Overall effect of group	Smoking	0.029						
Time x group		0.787[c]						
Baseline		Ref	79	7 (8.9)	-	76	8 (10.5)	-
Time 1		0.971	62	4 (6.5)	-	63	9 (14.2)	-
Time 3		0.309	48	5 (10.4)	-	52	9 (17.3)	-
Time 5		0.461	65	3 (4.6)	-	65	7 (10.8)	-

SWE-CES=Patient empowerment; GSES=General Self-Efficacy; EQ-VAS=health status; HDL-C=high-density lipoprotein cholesterol, mmol/L; BMI=Body Mass Index, kg/m2;
a=Interaction year x group *p*<.05. Analysis stratified by groups (PBL) and (Control group)
b=Interaction year x group *p*<.10. Analysis stratified by group (PBL) and (Control group)
c=Interaction year x group *p*>.10. Analysis not stratified
Time 1=One year follow-up
Time 3=Three years follow-up
Time 5=Five years follow-up

For peer review only

Main findings

SWE-CES

There was a statistically significant change over time of patient SWE-CES (patient empowerment) from baseline to time 5 ($p = 0.025$) in the total group of participants (Table 2).

EQ-VAS

We observed a statistically significant change of EQ-VAS (self-rated health status) from baseline to time 1 ($p = 0.038$). The interaction between time and group was significant at the level of 5% ($p = 0.022$). Therefore, the analysis was stratified by group. EQ-VAS increased significantly at time 1 ($M = 74.64 \pm 18.05$, $p = 0.026$), time 3 ($M = 78.27 \pm 14.84$, $p = 0.007$), and time 5 ($M = 77.33 \pm 15.52$, $p = 0.031$) in the PBL group compared to baseline, independently of negative effects of co-variates age ($p = 0.018$), while positive effects of low education increased 10 units compared to high education ($p = 0.011$). No significant changes were observed in the control group at time 1 ($M = 75.30 \pm 16.36$, $p = 0.501$), time 3 ($M = 71.89 \pm 18.30$, $p = 0.195$), and time 5 ($M = 72.06 \pm 17.19$, $p = 0.137$) compared to baseline (Table 2). The co-variate living alone showed a negative effect on EQ-VAS by 7.5 units compared with living together ($p = 0.039$).

HDL-C

There was a statistically significant change of HDL-C (high density lipoprotein cholesterol) from baseline to time 5 ($p = 0.036$) (Table 2). The interaction between time and group was significant at alpha level 5% ($p = 0.016$). Therefore, the analysis was stratified by group. HDL-C increased significantly at time 1 ($M = 1.37$ mmol/L ± 0.43, $p = 0.003$), time 3 ($M = 1.42$ mmol/L ± 0.55, $p < 0.001$), and time 5 ($M = 1.39$ mmol/L ± 0.41, $p < 0.001$) in the PBL group compared to baseline, independently of the positive effects of co-variates sex (woman) ($p = 0.002$) and negative effects of low education compared high education ($p = 0.035$). There was no significant change in the control group at time 1 ($M = 1.23 \pm 0.36$, $p = 0.781$), time 3

For peer review only - http://bmjopen.bmj.com/site/about/guidelines.xhtml

($M = 1.27 \pm 0.37$, $p = 0.655$) or time 5 ($M = 1.27 \pm 0.42$, $p = 0.459$) compared to baseline (Table 2). However, being a woman was positively associated with HDL-C ($p = 0.026$).

BMI

There was no statistically significant main effect (time or group) for BMI (Body Mass Index) (Table 2).

Weight

There was a significant interaction between time and group at alpha level of 5% ($p = 0.04$). Therefore, the analysis was stratified by group. No significant differences were observed between time 1 and 5 in the PBL group compared to baseline. However, the mean weight of patients in the control group at time 1 increased significantly compared to baseline ($p = 0.045$) (Table 2).

GSES

For GSES (self-efficacy) there was no significant group effect or change over time (Table 2).

Smoking

There was no significant interaction between time and intervention ($p = 0.79$), but there was a significant "group" effect ($p = 0.029$) for smoking, showing that the overall proportion of smokers in the control group was statistically significantly higher than the proportion of smokers in the PBL group (Table 2).

<u>Additional findings - post hoc sensitivity analysis with interaction test significant at 10%.</u>

SWE-CES

The interaction between time and group was ($p = 0.086$). This implies that the analysis of SWE-CES stratified by group showed a significant increase in the PBL group as a part of the sensitivity analysis. SWE-CES increased significantly at time 3 ($M = 38.3 \pm 5.7$, $p = 0.023$)

For peer review only - http://bmjopen.bmj.com/site/about/guidelines.xhtml

and time 5 (M = 39.3 ± 5.2, p <0.001) (Table 2) compared to the baseline, independently of positive effects of co-variates low education (p = 0.041); it did not, however, change significantly over time in the control group. Being a woman did, however, negatively effect SWE-CES (p = 0.010).

BMI

There was a significant interaction between time and group at the significance level 10% (p = 0.061). Therefore, the analysis was stratified by group. The analysis stratified by group showed a slight decrease from baseline (M = 27.12 ± 3.91) after 1-year (M = 26.83 ± 3.93, p = 0.076), but that did not remain over time. No significant changes over time were observed for the control group (Table 2).

Discussion

We investigated the long-term effects of a one-year PBL intervention in primary care on patient empowerment, self-efficacy, health status and cardiac risk factors in patients with CHD. PBL improved patient empowerment, health status and HDL-C over the 5 years follow-up compared to control group. However, PBL did not result in changes in BMI, weight, smoking or self-efficacy.

We observed significant changes from PBL in patient empowerment and health status over the five-year follow-up compared to the one-year follow-up study [14]. It seems possible that it might take a long time to adapt to longstanding behavioral changes. As many studies in this area often end within a year [4,5,29] our study indicates that long term follow-up is needed to understand the effects on patient behavioural outcomes of interventions using social cognitive theories.

Cardiac rehabilitation reduces risk factors but may also improve health over time [30].
A national prospective cohort study with nearly 4,500 participants showed that cardiac

For peer review only - http://bmjopen.bmj.com/site/about/guidelines.xhtml

rehabilitation had positive effects on quality of life up to one year after hospital discharge compared to non-participants [31]. In our study, health status improved up to one year, and also after three and five years compared to the control group. PBL allows patients to actively decide what is important to discuss in cardiac rehabilitation, suggesting that PBL might promote control over the disease, leading to better health.

We found that HDL-C improved over the five years follow-up. Similar results have been reported in short term follow-up studies on cardiac risk factors using combined education and written information intensive health education sessions and individual support [32,33]. These studies share a focus on education, but we have used PBL in group sessions in a period of one year. Our findings confirm that cardiac rehabilitation focusing on behavioral strategies may be beneficial in maintaining healthy levels of HDL-C.

Multidisciplinary weigh loss behavioral interventions in cardiac rehabilitation may be effective in the short run [34]. We did not observe any changes in BMI between or within groups over time. In contrast, a meta-analysis study involving almost 20,000 participants showed that internet-based education significantly reduced cardiac risk factors regarding blood pressure, blood lipids, weight and physical inactivity, with changes lasting up to one year [29].

A cognitive nurse-led intervention designed to improve physical activity by using repeated telephone calls and text messaging consultations improved BMI at a 6-month follow-up compared with controls [35]. Our study was also nurse-led, but the intervention did not include extra consultations, telephone calls or text message for achieving lifestyle goals. Digital aids with continuous support may be helpful when new health promotion activities are being started [36,37].

The results from the Euroaspire V study reported a high prevalence of persistent smokers after a CHD event [8]. In this study, PBL did not result in a successful lifestyle change in smoking

For peer review only - http://bmjopen.bmj.com/site/about/guidelines.xhtml

during the follow-up. Even though few participants were smokers in our study, there was a trend, albeit not a significant one, of participants in the control group being smokers more often than those in the PBL group. Higher age, low level of education and living alone were also significantly associated with smoking. As reported from other research, these socio-demographic determinants could be considered in stratifying for cardiac risk factors [38].

We observed no long-term effects of PBL on self-efficacy. This finding is in contrast to Su et al, who reported significant improvements in self-efficacy from an eHealth cardiac rehabilitation intervention [39]. Both studies included cardiac patients and used social cognitive theory methods. However, the inconsistency can probably be explained by the facts that participants in our study were older, several lived alone, and the majority had compulsory education. We accounted for these factors, suggesting that future studies may need to take sociodemographic factors into account in studies designed for cardiac rehabilitation interventions.

The COR-PRIM study has some study limitations and strengths that we would like to address. First, it is a challenge to manage problems arising due to loss of follow-up and deaths while performing a study with long-term follow up design. Secondly, an increased patient empowerment (SWE-CES) in patients may not result in adherence to guidelines. Most of the patients were retired and co-habiting, which may imply that lifestyles were influenced by partners [40]. They were invited to some of the PBL sessions to take part in discussions about their own questions with health care professionals, but we did not follow up the spouses after the intervention. However, the PBL-intervention was performed for one year, and we believe this is a strength, as the education became a part of the patients' lives.

A major strength of our study is the long-term follow-up, which is very much needed in interventions aiming at cardiac preventive behavioral changes. Another strength is that the intervention was led by pedagogical trained specialist nurses who worked in primary care. A

For peer review only - http://bmjopen.bmj.com/site/about/guidelines.xhtml

further advantage is that patients could choose to participate in group education, but few chose to participate, a web-based PBL education may therefore be a future option.

These findings suggest that patients participating in a one-year PBL programme may strengthen patient empowerment and health status as well as reducing cardiac risk factors. PBL education aiming to improve patient empowerment in cardiac rehabilitation may be possible to apply in other primary care settings.

Conclusion

One-year PBL intervention has positive effects on patient empowerment, health status and HDL-C at a five-year follow-up. Further experimental studies would have to consider sociodemographic factors to improve self-management and cardiac risk factors in CHD.

Acknowledgments: We would like to thank study participants and nurses who contributed to the PBL intervention and for the monitoring during the follow-ups.

Contributors: CA, PT, SN, TJ and AKK contributed to the conception or design of the work. AKK contributed to the acquisition of the work and CA, PT, SN, TJ, NK and AKK contributed to analysis or interpretation of data for the work. CA drafted the manuscript and AKK, PT, SN, TJ, NK made substantial intellectual contributions to the manuscript. All authors agree to be accountable for all parts of the manuscript and approve the publication of the final manuscript.

Funding: This work was supported by the Swedish Heart and Lung Association project numbers E091/10, E122/11, E083/12, and E103/13 and the Country Council of Region Östergötland, Sweden, project numbers LIO-92281, − 125151, − 27535, − 354951, and LIO-433801.

Competing interests: None declared

For peer review only - http://bmjopen.bmj.com/site/about/guidelines.xhtml

Patient and public involvement: Patients and/or the public were not involved in the design, or conduct, or reporting, or dissemination plans of this research.

Patients consent for publication: Consent obtained directly from patients.

Ethics approval: This study involves human participants and was approved by Regional Ethics Committee of Linköping, Dnr 2010/128-31. Participants gave informed consent to participate in the study before taking part.

Provenance and peer review: Not commissioned; externally peer reviewed.

Data sharing statement: Authors declare that they are willing to share study data on reasonable request.

For peer review only

For peer review only - http://bmjopen.bmj.com/site/about/guidelines.xhtml

References

1. Timmis A, Townsend N, Gale CP, *et al.* European Society of Cardiology: Cardiovascular Disease Statistics 2019. *Eur Heart J* 2020;41:12-85.doi:10.1093/eurheartj/ehz859

2. WHO. Global Health Estimates 2020: Deaths by Cause, Age, Sex, by Country and by Region, 2000-2019. Geneva, World Health Organization; 2020. https://www.who.int/data/gho/data/themes/mortality-and-global-health-estimates (accessed 26 May 2022)

3. Ambrosetti M, Abreu A, Corra U, *et al.* Secondary prevention through comprehensive cardiovascular rehabilitation: From knowledge to implementation. 2020 update. A position paper from the Secondary Prevention and Rehabilitation Section of the European Association of Preventive Cardiology. *Eur J Prev Cardiol* 2020;10.1177/2047487320913379:2047487320913379.doi:10.1177/2047487320913379

4. Mills M, Loney P, Jamieson E, *et al.* A primary care cardiovascular risk reduction clinic in Canada was more effective and no more expensive than usual on-demand primary care – a randomised controlled trial. *Health SoC Care Community* 2010;18:30-40.doi:10.1111/j.1365-2524.2009.00872.x

5. Storm V, Dorenkamper J, Reinwand DA, *et al.* Effectiveness of a Web-Based Computer-Tailored Multiple-Lifestyle Intervention for People Interested in Reducing their Cardiovascular Risk: A Randomized Controlled Trial. *J Med Internet Res* 2016;18:e78.doi:10.2196/jmir.5147

6. Goyer L, Dufour R, Janelle C, *et al.* Randomized controlled trial on the long-term efficacy of a multifaceted, interdisciplinary lifestyle intervention in reducing cardiovascular risk and improving lifestyle in patients at risk of cardiovascular disease. *J Behav Med* 2013;36:212-224.doi:10.1007/s10865-012-9407-3

For peer review only

7. Ferrières J, Lautsch D, Ambegaonkar BM*, et al.* Use of guideline-recommended management in established coronary heart disease in the observational DYSIS II study. *Int J Cardiol* 2018;270:21-27.doi:10.1016/j.ijcard.2018.06.008

8. Kotseva K, De Backer G, De Bacquer D*, et al.* Lifestyle and impact on cardiovascular risk factor control in coronary patients across 27 countries: Results from the European Society of Cardiology ESC-EORP EUROASPIRE V registry. *Eur J Prev Cardiol* 2019;26:824-835.doi:10.1177/2047487318825350

9. Olsen SJ, Schirmer H, Bonaa KH*, et al.* Cardiac rehabilitation after percutaneous coronary intervention: Results from a nationwide survey. *Eur J Cardiovasc Nurs* 2018;17:273-279.doi:10.1177/1474515117737766

10. Gomez-Velasco DV, Almeda-Valdes P, Martagon AJ*, et al.* Empowerment of patients with type 2 diabetes: current perspectives. *Diabetes Metab Syndr Obes* 2019;12:1311-1321.doi:10.2147/DMSO.S174910

11. Williams B, Pace AE. Problem based learning in chronic disease management: A review of the research. *Patient Educ Couns* 2009;77:14-19.doi:10.1016/j.pec.2009.03.004

12. Karner A, Nilsson S, Jaarsma T*, et al.* The effect of problem-based learning in patient education after an event of CORONARY heart disease--a randomised study in PRIMARY health care: design and methodology of the COR-PRIM study. *BMC Fam Pract* 2012;13:110.doi:10.1186/1471-2296-13-110

13. Lilly CL, Bryant LL, Leary JM*, et al.* Evaluation of the effectiveness of a problem-solving intervention addressing barriers to cardiovascular disease prevention behaviors in 3 underserved populations: Colorado, North Carolina, West Virginia, 2009. *Prev Chronic Dis* 2014;11:E32.doi:10.5888/pcd11.130249

For peer review only

14. Köhler AK, Jaarsma T, Tingström P*, et al.* The effect of problem-based learning after coronary heart disease - a randomised study in primary health care (COR-PRIM). *BMC Cardiovasc Disord* 2020;20:370.doi:10.1186/s12872-020-01647-2

15. Ogmundsdottir Michelsen H, Sjolin I, Schlyter M*, et al.* Cardiac rehabilitation after acute myocardial infarction in Sweden - evaluation of programme characteristics and adherence to European guidelines: The Perfect Cardiac Rehabilitation (Perfect-CR) study. *Eur J Prev Cardiol* 2020;27:18-27.doi:10.1177/2047487319865729

16. Leksell J, Funnell M, Sandberg G*, et al.* Psychometric properties of the Swedish Diabetes Empowerment Scale. *Scand J Caring Sci* 2007;21:247-252.doi:10.1111/j.1471-6712.2007.00463.x

17. Gadermann AM, Zumbo BD, Zeisser C. Ordinal versions of coefficients alpha and theta for likert rating scales. *J Mod Appl Stat Methods* 2007;6:21-29.doi:10.22237/jmasm/1177992180

18. Löve J, Moore Crystal D, Hensing G. Validation of the Swedish translation of the general self-efficacy scale. *Qual Life Res* 2012;21:1249-1253.doi:

19. Tanimura C, Aoto H, Kobayashi N*, et al.* Effects of a Self-efficacy Theory-Based Training Program for Peers of Patients with Type 2 Diabetes. *Yonago Acta Med* 2020;63:282-293.doi:10.33160/yam.2020.11.006

20. Tan CCL, Hwang SW, Zhang N*, et al.* Effect of a Diabetes Self-Efficacy Enhancing Program on Older Adults With Type 2 Diabetes: A Randomized Controlled Trial. *Clin Nurs Res* 2020;29:293-303.doi:10.1177/1054773818792480

21. Tang CY, Blackstock FC, O'Halloran PD*, et al.* A Standard Form of Cardiac Rehabilitation Can Improve Self-Reported Positive Behavior Changes in the Short- to Medium-Term. *J Cardiopulm Rehabil* 2019;39:175-180.doi:10.1097/HCR.0000000000000371

For peer review only - http://bmjopen.bmj.com/site/about/guidelines.xhtml

22. Borzou SR, Amiri S, Salavati M, *et al.* Effects of the First Phase of Cardiac Rehabilitation Training on Self-Efficacy among Patients Undergoing Coronary Artery Bypass Graft Surgery. *J Tehran Univ Heart Center* 2018;13:126-131.

23. Madueño-Caro AJ, Pardos-Lafarga M, Muñoz-Ayllón M, *et al.* Cardiac rehabilitation in primary care. Impact of an intervention on perceived self-efficacy. *J Funct Morphol.* 2019;410.3390/jfmk4020032

24. Lenz ER, Shortridge-Baggett LM. Self efficacy in nursing. research and measurement perspectives: Springer Pub 2002.

25. EuroQol Research Foundation. EQ-5D-Y User Guide. https://euroqol.org/publications/user-guides/ (accessed 26 May 2022).

26. Cheng LJ, Tan RL, Luo N. Measurement Properties of the EQ VAS Around the Globe: A Systematic Review and Meta-Regression Analysis. *Value Health* 2021;24:1223-1233.doi:10.1016/j.jval.2021.02.003

27. Gurka MJ, Filipp SL, Musani SK, *et al.* Use of BMI as the marker of adiposity in a metabolic syndrome severity score: Derivation and validation in predicting long-term disease outcomes. *Metab Clin Exp* 2018;83:68-74.doi:10.1016/j.metabol.2018.01.015

28. Gibson RS. Principles of nutritional assessment. New York: Oxford University Press 2005.

29. Beishuizen CR, Stephan BC, van Gool WA, *et al.* Web-Based Interventions Targeting Cardiovascular Risk Factors in Middle-Aged and Older People: A Systematic Review and Meta-Analysis. *J Med Internet Res* 2016;18:e55.doi:10.2196/jmir.5218

30. Hansen VB, Maindal HT. Cardiac rehabilitation with a nurse case manager (GoHeart) across local and regional health authorities improves risk factors, self-care and psychosocial outcomes. A one-year follow-up study. *JRSM Cardiovascular Disease* 2014;3:1-11.doi:10.1177/2048004014555922

For peer review only - http://bmjopen.bmj.com/site/about/guidelines.xhtml

31. Hurdus B, Munyombwe T, Dondo TB*, et al.* Association of cardiac rehabilitation and health-related quality of life following acute myocardial infarction. *Heart (British Cardiac Society)* 2020;106:1726-1731.doi:10.1136/heartjnl-2020-316920

32. Dehghani A, Kumar Bhasin S, Dwivedi S*, et al.* Influence of Comprehensive Life Style Intervention in Patients of CHD. *Glob J Health Sci* 2015;7:6-16.doi:10.5539/gjhs.v7n7p6

33. Salas-Salvadó J, Díaz-López A, Basora J*, et al.* Effect of a lifestyle intervention program with energy-restricted Mediterranean diet and exercise on weight loss and cardiovascular risk factors: One-year results of the PREDIMED-Plus trial. *Diabetes Care* 2019;42:777-788.doi:10.2337/dc18-0836

34. Gallagher R, Kirkness A, Armari E*, et al.* Weight management issues and strategies for people with high cardiovascular risk undertaking an Australian weight loss program: a focus group study. *Nurs Health Sci* 2012;14:18-24.doi:10.1111/j.1442-2018.2011.00651.x

35. Alsaleh E, Windle R, Blake H. Behavioural intervention to increase physical activity in adults with coronary heart disease in Jordan. *BMC Public Health* 2016;16:643.doi:10.1186/s12889-016-3313-5

36. Karyotaki E, Ebert DD, Donkin L*, et al.* Do guided internet-based interventions result in clinically relevant changes for patients with depression? An individual participant data meta-analysis. *Clin Psychol Rev* 2018;63:80-92.doi:10.1016/j.cpr.2018.06.007

37. Johansson P, Westas M, Andersson G*, et al.* An Internet-Based Cognitive Behavioral Therapy Program Adapted to Patients With Cardiovascular Disease and Depression: Randomized Controlled Trial. *JMIR Ment Health* 2019;6:e14648.doi:10.2196/14648

38. Safford MM, Reshetnyak E, Sterling MR*, et al.* Number of Social Determinants of Health and Fatal and Nonfatal Incident Coronary Heart Disease in the REGARDS Study. *Circulation* 2021;143:244-253.doi:10.1161/circulationaha.120.048026

For peer review only - http://bmjopen.bmj.com/site/about/guidelines.xhtml

39. Su JJ, Yu DS. Effects of a nurse-led eHealth cardiac rehabilitation programme on health outcomes of patients with coronary heart disease: A randomised controlled trial. *Int J Nurs Stud* 2021;122:104040.doi:10.1016/j.ijnurstu.2021.104040

40. Köhler AK, Nilsson S, Jaarsma T, *et al.* Health beliefs about lifestyle habits differ between patients and spouses 1 year after a cardiac event - a qualitative analysis based on the Health Belief Model. *Scand J Caring Sci* 2017;31:332-341.doi:10.1111/scs.12351

For peer review only

Figure Legend:

Figure 1. Flowchart on study participation.

Figure 2. Distribution of outcome variables in PBL and control groups at baseline 1, 3 and 5 year follow-up.

For peer review only - http://bmjopen.bmj.com/site/about/guidelines.xhtml

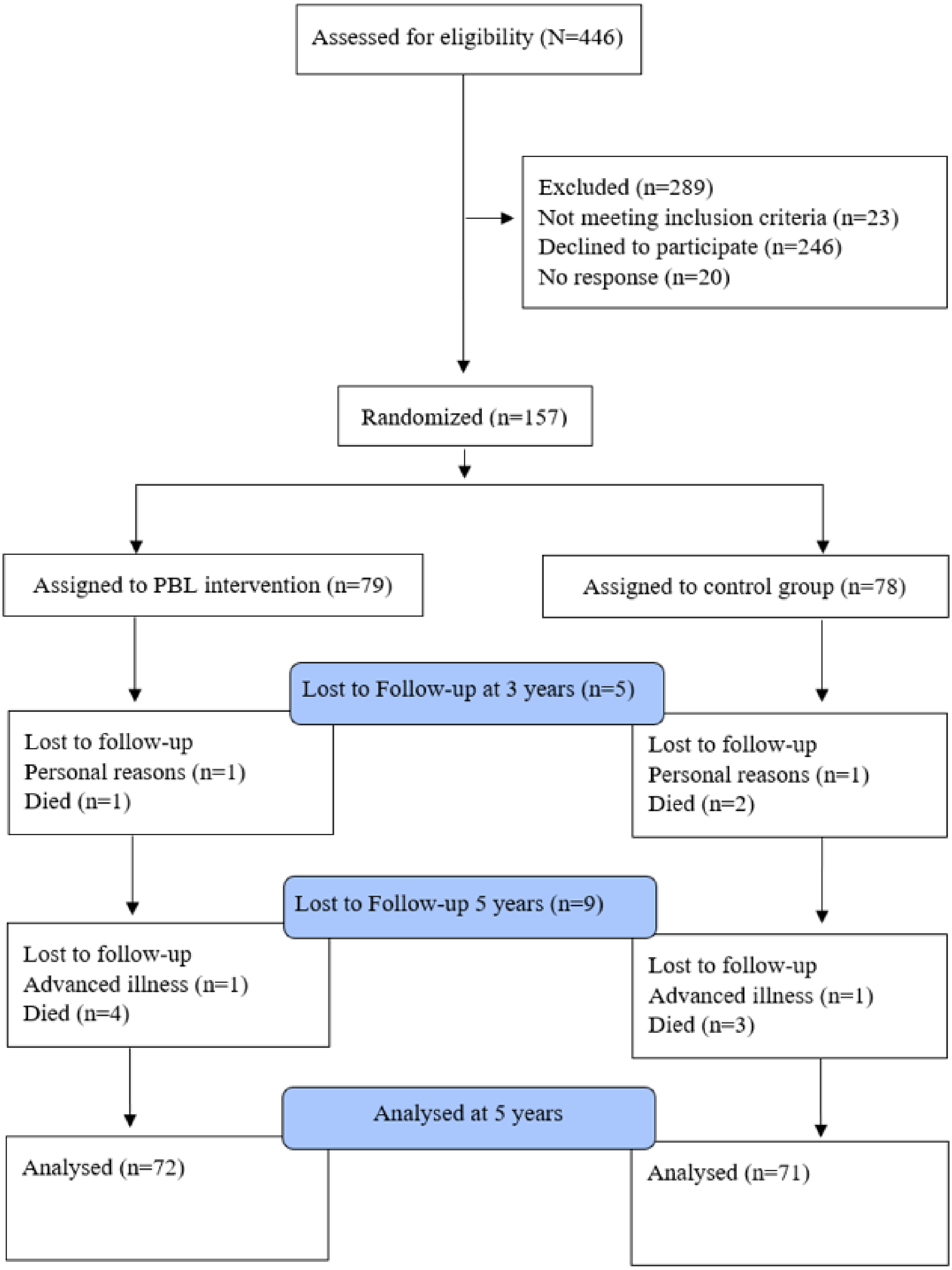

Figure 1. Flowchart on study participation

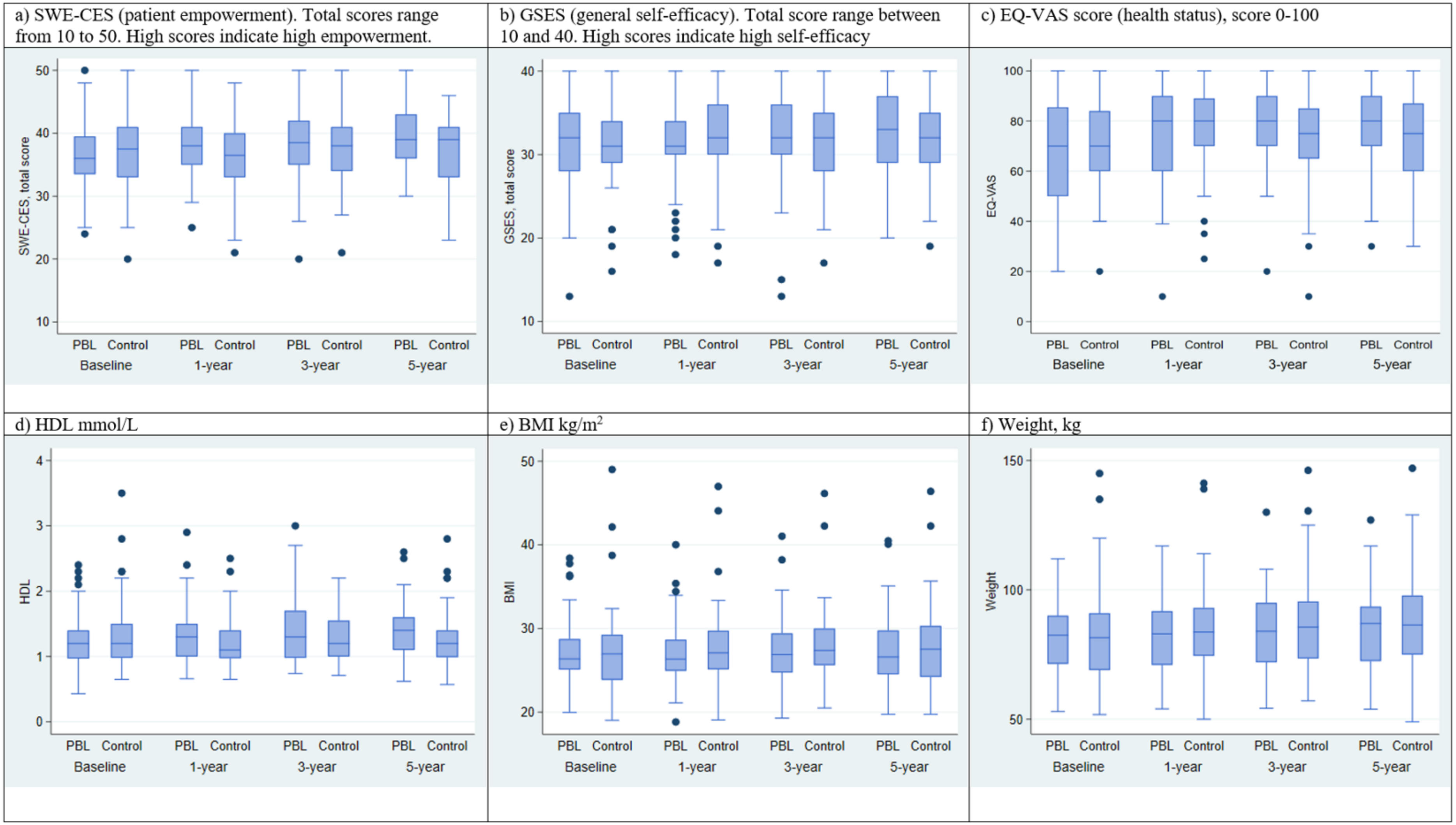

Figure 2. Distribution of outcome variables in PBL and control groups at baseline 1, 3 and 5 year follow-up.

CONSORT 2010 checklist of information to include when reporting a randomised trial*

Section/Topic	Item No	Checklist item	Reported on page No
**Title and abstract**			
	1a	Identification as a randomised trial in the title	1
	1b	Structured summary of trial design, methods, results, and conclusions (for specific guidance see CONSORT for abstracts)	2,3
**Introduction**			
Background and objectives	2a	Scientific background and explanation of rationale	4,5
	2b	Specific objectives or hypotheses	5
**Methods**			
Trial design	3a	Description of trial design (such as parallel, factorial) including allocation ratio	5,6
	3b	Important changes to methods after trial commencement (such as eligibility criteria), with reasons	NA
Participants	4a	Eligibility criteria for participants	6
	4b	Settings and locations where the data were collected	6
Interventions	5	The interventions for each group with sufficient details to allow replication, including how and when they were actually administered	7+ Figure 1
Outcomes	6a	Completely defined pre-specified primary and secondary outcome measures, including how and when they were assessed	8
	6b	Any changes to trial outcomes after the trial commenced, with reasons	NA
Sample size	7a	How sample size was determined	7
	7b	When applicable, explanation of any interim analyses and stopping guidelines	NA
Randomisation:			
Sequence generation	8a	Method used to generate the random allocation sequence	6
	8b	Type of randomisation; details of any restriction (such as blocking and block size)	6
Allocation concealment mechanism	9	Mechanism used to implement the random allocation sequence (such as sequentially numbered containers), describing any steps taken to conceal the sequence until interventions were assigned	6
Implementation	10	Who generated the random allocation sequence, who enrolled participants, and who assigned participants to interventions	6
Blinding	11a	If done, who was blinded after assignment to interventions (for example, participants, care providers, those	6

For peer review only

		assessing outcomes) and how	
	11b	If relevant, description of the similarity of interventions	NA
Statistical methods	12a	Statistical methods used to compare groups for primary and secondary outcomes	9,10
	12b	Methods for additional analyses, such as subgroup analyses and adjusted analyses	10
**Results**			
Participant flow (a diagram is strongly recommended)	13a	For each group, the numbers of participants who were randomly assigned, received intended treatment, and were analysed for the primary outcome	Figure 1
	13b	For each group, losses and exclusions after randomisation, together with reasons	Figure 1
Recruitment	14a	Dates defining the periods of recruitment and follow-up	6
	14b	Why the trial ended or was stopped	NA
Baseline data	15	A table showing baseline demographic and clinical characteristics for each group	Table 1
Numbers analysed	16	For each group, number of participants (denominator) included in each analysis and whether the analysis was by original assigned groups	Table 2
Outcomes and estimation	17a	For each primary and secondary outcome, results for each group, and the estimated effect size and its precision (such as 95% confidence interval)	NA
	17b	For binary outcomes, presentation of both absolute and relative effect sizes is recommended	NA
Ancillary analyses	18	Results of any other analyses performed, including subgroup analyses and adjusted analyses, distinguishing pre-specified from exploratory	16 + Table 2
Harms	19	All important harms or unintended effects in each group (for specific guidance see CONSORT for harms)	NA
**Discussion**			
Limitations	20	Trial limitations, addressing sources of potential bias, imprecision, and, if relevant, multiplicity of analyses	3,19
Generalisability	21	Generalisability (external validity, applicability) of the trial findings	20
Interpretation	22	Interpretation consistent with results, balancing benefits and harms, and considering other relevant evidence	17-19
**Other information**			
Registration	23	Registration number and name of trial registry	6
Protocol	24	Where the full trial protocol can be accessed, if available	Ref 12
Funding	25	Sources of funding and other support (such as supply of drugs), role of funders	21

*We strongly recommend reading this statement in conjunction with the CONSORT 2010 Explanation and Elaboration for important clarifications on all the items. If relevant, we also recommend reading CONSORT extensions for cluster randomised trials, non-inferiority and equivalence trials, non-pharmacological treatments, herbal interventions, and pragmatic trials. Additional extensions are forthcoming: for those and for up to date references relevant to this checklist, see www.consort-statement.org.

For peer review only - http://bmjopen.bmj.com/site/about/guidelines.xhtml

BMJ Open

Does problem-based learning improve patient empowerment and cardiac risk factors in patients with coronary heart disease in a Swedish primary care setting? A long-term prospective, randomized, parallel single randomized trial (COR-PRIM).

Journal:	*BMJ Open*
Manuscript ID	bmjopen-2022-065230.R1
Article Type:	Original research
Date Submitted by the Author:	11-Nov-2022
Complete List of Authors:	Andreae, Christina; Linkoping University Department of Health Medicine and Caring Sciences,<br>Tingström, Pia; Linkoping University Department of Health Medicine and Caring Sciences<br>Nilsson, Staffan; Linkoping University Department of Health Medicine and Caring Sciences<br>Jaarsma, Tiny; Linkoping University Department of Health Medicine and Caring Sciences<br>Karlsson, Nadine; Linkoping University Department of Health Medicine and Caring Sciences<br>Köhler, Anita Kärner; Linkoping University Department of Health Medicine and Caring Sciences
**Primary Subject Heading**:	Cardiovascular medicine
Secondary Subject Heading:	Medical education and training
Keywords:	Coronary heart disease < CARDIOLOGY, PRIMARY CARE, EDUCATION & TRAINING (see Medical Education & Training)

SCHOLARONE™
Manuscripts

I, the Submitting Author has the right to grant and does grant on behalf of all authors of the Work (as defined in the below author licence), an exclusive licence and/or a non-exclusive licence for contributions from authors who are: i) UK Crown employees; ii) where BMJ has agreed a CC-BY licence shall apply, and/or iii) in accordance with the terms applicable for US Federal Government officers or employees acting as part of their official duties; on a worldwide, perpetual, irrevocable, royalty-free basis to BMJ Publishing Group Ltd ("BMJ") its licensees and where the relevant Journal is co-owned by BMJ to the co-owners of the Journal, to publish the Work in this journal and any other BMJ products and to exploit all rights, as set out in our licence.

The Submitting Author accepts and understands that any supply made under these terms is made by BMJ to the Submitting Author unless you are acting as an employee on behalf of your employer or a postgraduate student of an affiliated institution which is paying any applicable article publishing charge ("APC") for Open Access articles. Where the Submitting Author wishes to make the Work available on an Open Access basis (and intends to pay the relevant APC), the terms of reuse of such Open Access shall be governed by a Creative Commons licence – details of these licences and which Creative Commons licence will apply to this Work are set out in our licence referred to above.

Other than as permitted in any relevant BMJ Author's Self Archiving Policies, I confirm this Work has not been accepted for publication elsewhere, is not being considered for publication elsewhere and does not duplicate material already published. I confirm all authors consent to publication of this Work and authorise the granting of this licence.

For peer review only

Does problem-based learning improve patient empowerment and cardiac risk factors in patients with coronary heart disease in a Swedish primary care setting? A long-term prospective, randomized, parallel single randomized trial (COR-PRIM).

Christina Andreae[1,2], Pia Tingström[1], Staffan Nilsson[3], Tiny Jaarsma[1,4] Nadine Karlsson[1] and Anita Kärner Köhler[1]

1. Department of Health, Medicine and Caring Sciences, Linköping University, Linköping, Sweden.

2. Centre for Clinical Research Sörmland, Uppsala University, Eskilstuna, Sweden.

3. Primary Health Care Center Vikbolandet, and Department of Health, Medicine and Caring Sciences, Linköping University, Linköping, Sweden.

4. Julius Center, University Medical Center Utrecht, Utrecht, the Netherlands.

Corresponding author: Christina Andreae, PhD, RN, Department of Medicine, Region Sörmland, 63188 Eskilstuna, Sweden

Email: christina.andreae@liu.se

Tel: +4616 10 44 07

For peer review only

Abstract

Objectives: To investigate long-term effects of a one-year problem-based learning (PBL) on self-management and cardiac risk factors in patients with coronary heart disease (CHD).

Design: A prospective, randomized, parallel single centre trial.

Settings: Primary care settings in Sweden.

Participants: 157 patients with stable CHD completed the study. Subjects with reading and writing impairments, mental illness or expected survival less than one year were excluded.

Intervention: Participants were randomized and assigned to receive either PBL (intervention) or home-sent patient information (control group). In this study participants were followed-up at baseline 1, 3 and 5 years.

Primary and secondary outcomes: Primary outcome was patient empowerment (SWE-CES) and secondary outcomes general self-efficacy (GSES), self-rated health status (EQ-VAS), high-density lipoprotein cholesterol (HDL-C), Body Mass Index (BMI), weight and smoking. Outcomes were adjusted for sociodemographic factors.

Results: The PBL intervention group resulted in a significant improved change in SWE-CES over the five-year period (Mean [M], 39.39; 95% confidence interval [CI], 37.88-40.89) compared to the baseline (M, 36.54; CI, 35.40-37.66). PBL intervention group increased HDL-C level (M, 1,39; CI, 1.28-1.50) compared to baseline (M, 1,24; CI, 1.15-1.33) and for EQ-VAS (M, 77.33; CI, 73.21-81.45) compared to baseline (M, 68.13; CI, 63.66-72.59) while these outcomes remained unchanged in the control group. There were no significant differences in BMI, weight, or scores on GSES, neither between nor within groups over time. The overall proportion of smokers was significantly higher in the control group than in the experimental group.

Conclusion: One-year PBL intervention had positive effect on patient empowerment, health status and HDL-C at a five-year follow-up compared to the control group. PBL education

For peer review only - http://bmjopen.bmj.com/site/about/guidelines.xhtml

aiming to improve patient empowerment in cardiac rehabilitation should account for sociodemographic factors.

Keywords: Coronary disease, empowerment, heart disease risk factors, long-term, primary health care, problem-based learning, randomized controlled trial.

Trial registration: The study was registered at ClinicalTrial.gov with number NCT01462799.

Strengths and limitations of this study:

- The majority of participants remained in the study over the 5-years follow-up.
- Baseline measurement was well balanced for both groups regarding sociodemographic and study variables.
- The PBL intervention was held by a small group of specialist nurses who were advanced trained in PBL.
- Participants were most retired, were men and lived in sub-urban areas which need to be considered in the implementation of PBL in cardiac primary care settings.

Introduction

Nearly 126 million people globally suffer from coronary heart disease (CHD), the leading cause of death worldwide [1,2]. Important treatment goals include slowing down the underlying atherosclerosis process by targeting hypertension, high cholesterol, diabetes, overweight, tobacco use, alcohol consumption, physical inactivity, and poor diet [3]. Cardiac rehabilitation in post-myocardial infarction improve lifestyle habits such as physical activity and dietary intake, and has also shown positive effects on blood lipids, blood pressure and smoking [4-6]. In

For peer review only - http://bmjopen.bmj.com/site/about/guidelines.xhtml

For peer review only

a small randomized trial, cardiac rehabilitation programme for patients with myocardial infarction or who had undergone coronary artery by-pass graft surgery (CABG), focusing on stress management, physical exercise and dietary intake, reduced morbidity and hospitalization for CHD [7]. Attendance in cardiac rehabilitation programme has long-term effects on survival among patients who have undergone CABG with reduced 10-year all-cause mortality [8]. Cardiac rehabilitation enables patients to make lifestyle changes that are important for maintaining health. Cardiac rehabilitation consists of multidisciplinary interventions where patients are offered tailored education and counselling with the goals of improving health behavior for sustainable secondary prevention [3]. Several studies have investigated the effect of interventions such as nurse telephone follow-up or group education, and described the effects on illness perception, self-efficacy, behaviour change and cardiac risk factors [9-11]. The effects of several programme on cardiac risk factors or behaviour were found from 3 months up to 2 years. Patients' attendance of cardiac rehabilitation appears to be low, and risk factors remain or even deteriorate from the first to second cardiac event [12-14]. For example, persistent smoking is common after discharge from hospital, and intentions to quit smoking in the near future remain low. This regards also overweight and physical activity after a cardiac event, many patients do not lose weight and nearly half of patients perform no physical activity and do not make changes to their physical status [13]. Thus, primary care has great challenges motivating patients to achieve healthy lifestyles.

Empowering patients to take control over the disease, and making them aware of factors that affect illness, have positively impacted on health outcomes in chronic diseases [15]. Problem-based learning (PBL) is a method that empowers patients to become aware of how to reduce risk factors in chronic diseases [16]. PBL is a cognitive educational model that promotes self-learning where critical and reflective thinking are important components of learning outcomes and enhanced self-management. PBL is process-oriented, meaning that learning skills are

For peer review only - http://bmjopen.bmj.com/site/about/guidelines.xhtml

developed through active, creative, and cognitive processes. Previous knowledge is placed in the light of real situations where knowledge is developed in a context together with others, all led by a trained PBL tutor [17]. A focus group intervention with problem solving intervention in a cardiac population showed that an eight-week focus group sessions resulted in significant improvements of stress management, dietary intake and physical activity [18]. The first results from a one-year follow-up in a coronary heart disease in primary care study (COR-PRIM) showed no differences in patient empowerment or self-efficacy between PBL intervention and patients who received home-sent patient information (controls). However, significant differences in secondary outcomes – i.e., body mass index, body weight and high-density lipoprotein cholesterol (HDL-C) – were found between the groups, with the PBL group performing favourably [19].

Although cardiac rehabilitation in Sweden has recently been assessed to be high quality [20] participation remains low. Only a few interventions incorporating a holistic view of health compromising psychological- and social aspects of health behaviors have been performed on the group level. PBL may empower self-management. The present study is elaborated in accordance with the COR-PRIM study basic aim, which was to discover whether PBL provided in primary health care for one year has long-term effects on patient empowerment and self-care, assessed at baseline and, at one, three, and five years after randomization. In this five year- and final assessment we wanted to identify if the findings in the 1-year follow-up remained or changed [19]. By this performance, this article is examining the sustainability of the effects by PBL, which to our knowledge has not been performed before.

Thus, the purpose of this study was to investigate the long-term effects on patient empowerment, self-efficacy, health status and cardiac risk factors in patients with CHD of a one-year PBL intervention in primary care, compared to home-sent patient information.

For peer review only

For peer review only - http://bmjopen.bmj.com/site/about/guidelines.xhtml

Methods

Design, sample and procedure

The COR-PRIM study was a prospective, randomized, parallel, and single centre trial designed to investigate whether one year of a PBL programme had long-term effects on patient empowerment, self-efficacy and cardiac risk factors [17]. The study protocol is registered at ClinicalTrial.gov, with the registration number NCT01462799. Patients diagnosed with CHD verified by percutaneous coronary intervention (PCI) or coronary artery by-pass surgery (CABG) and CABG+PCI or myocardial infarction within 6-12 months prior to the intervention and who had previously completed cardiac rehabilitation were eligible for the study. Additional criteria were that patients were stable in their heart disease, pharmacologically optimized in the last month before inclusion and, if applicable had completed cardiac school in the clinic. Patients with impaired ability to communicate and read in Swedish, verified psychiatric illness or short expected survival (of less than one year) were excluded [17]. The recruitment started in November 2011 and assessments of primary and secondary outcomes were collected until November 2019. Nurses at outpatient cardiac clinics identified eligible patients through medical records. These patients were then invited to participate in the study at a regular nurse-led cardiac follow-up visit. Patients received oral and written study information from a research assistant, and written consent was obtained from those interested in participating.

The study randomization and intervention

In COR-PRIM, a total of 157 patients were randomized and assigned to either problem-based learning intervention (PBL) or home-sent patient information (control group). The randomization procedure was based on block randomization with 18 study numbers based on a minimum of 12-18 study participants. Block randomization was performed using sealed and

For peer review only - http://bmjopen.bmj.com/site/about/guidelines.xhtml

unmarked envelopes. An assistant, blinded to the randomization and located outside the research setting, randomly allocated patients to either the PBL group (experimental) or home-sent patient information (control group) at a 1:1 ratio [17]. Nurses who performed the PBL intervention were not blinded, as the intervention was based on problem-based learning, which is different from regular care. The PBL intervention consisted of group learning sessions in primary care led by nurses who had advanced training in leading problem-based learning. Tutoring groups consisted of 6-9 participants and participated in 13 sessions (2 hours per meeting) over one year. This was based on the multiple of areas to discuss for lifestyles changes in CHD. For further information about the PBL intervention see Supplemental Material 1. Lifestyles changes require work over time and to be feasible in the patient's life, a long training is required. The PBL model has been validated as feasible in clinical settings [21]. Patients randomized to the control group received home-sent patient information reflecting a cognitive intervention, but did not participate in critical or reflective learning. Group effects were assessed after 11 sessions over one year [17].

Patient and public involvement

There were no patient or public involvement in the study.

Sample size

Sample size was calculated based on the primary outcome variable i.e., patient empowerment. The expected means for the Swedish Coronary Empowerment Scale (SWE-CES) were 30 and 36 for the control group and the PBL group respectively. With an estimated significance level of α=5% and a power of 1-β=80%, the sample size resulted in a minimum of 63 participants in each group [17]. Analyses conducted for the five-year follow-up period was n=72 (PBL) and n=71 (control group) (Figure 1).

Primary and secondary outcome measures

Patient empowerment was the study's primary outcome and was assessed at baseline, 1, 3 and

For peer review only - http://bmjopen.bmj.com/site/about/guidelines.xhtml

5 years (i.e., baseline, time 1, time 3 and time 5 respectively). Secondary outcomes included general self-efficacy while five secondary outcomes were added post hoc and these were health status, high-density lipoprotein cholesterol, body mass index, weight and smoking and were assessed at baseline, 1, 3 and 5 years.

Measurement

Sociodemographic variables and covariates

Sociodemographic variables consisting of age, sex, education level, marital status, place of residence, employment and smoking were determined by self-reported questionnaires. The covariates consisted of age, sex, education level and marital status.

Self-reported measures

The Swedish coronary empowerment scale (SWE-CES) measures how patients achieve goals, overcome barriers for goals achievement, and use the strategies necessary to make self-care choices. The strategies include dimensions of coping in managing disease, stress and dissatisfaction and also readiness to make health changes. The instrument contains 10 items that have 5 response alternatives ranged by Likert type options from 1 to 5. The total score ranges between 10 and 50, where high scores imply high level of patient empowerment. Four subscales measure different aspects of patient empowerment [22]. In this study, the total scores of the SWE-CES scale were used. Internal consistency reliability indicated acceptable values with Cronbach's alfa 0.751 [23].

Self-efficacy was measured by using the general self-efficacy scale (GSES). The instrument consists of 10 items that measure a person's belief in their own ability to implement behavioral changes in order to reduce risk factors for unhealthy lifestyles. Items are rated on a four-point Likert type scale ranging from 1 (not at all true) to 4 (exactly true). The total score ranges from 10 to 40, where a high total score indicates higher general self-efficacy [24]. Self-efficacy has been used to evaluate interventions to strengthen the self-care capacity of patients

For peer review only - http://bmjopen.bmj.com/site/about/guidelines.xhtml

with various chronic diseases such as diabetes [25,26] and patients with CHD [27-29]. It has been translated and psychometrically evaluated among a general Swedish population [30]. In this study, internal consistency reliability indicated acceptable values with Cronbach's alfa 0.914.

Health status

Self-rated health was assessed using EuroQol (EQ-VAS). The scale represents patients' overall health status and consists of a score between 0 and 100, where 0 indicates worst health and 100 the best health [31]. EQ-VAS has shown acceptable construct validity across populations [32].

Cardiac risk factors

High density lipoprotein cholesterol (HDL-C) was assessed using blood samples collected and analysed according to normal clinical routines. Anthropometric measurements of body size included length, weight, and Body Mass Index (BMI). Length and weight were measured in light cloths and with shoes removed. BMI is a widely used clinical measure and is recommended as indicator for defining obesity in adults. It is, furthermore, a reliable anthropometric measure in predicting metabolic syndromes [1,33]. BMI was calculated by dividing weight by the square of height in meters using the formula (weight (kg) / height (m^2)) [34]. Tobacco use was self-reported at each measure point.

Statistical analysis

The descriptive statistics included mean standard deviation (SD) or frequencies percent (%). Independent sample t-tests or Chi-square tests were used to analyses differences in sociodemographic and data characteristics between groups. Because the occasions (baseline to five-year) are nested within individuals, we employed a two-level mixed linear model with occasions ("time" at level 1) that are nested within individuals (level 2) to analyze continuous outcomes. The random part of the variance components model is individuals. The normality of continuous study outcomes was assessed using Shapiro-Wilk test. If the outcomes were

For peer review only - http://bmjopen.bmj.com/site/about/guidelines.xhtml

non-normally distributed, they were log transformed prior to the analysis. Smoking is a binary outcomes and was analysed with a logistic mixed model. The statistical analysis of each study outcome was performed using a mixed linear model with treatment group, age and sex as fixed variables, with occasions nested within individuals, and an interaction of time by treatment group. The interaction (combined effect) between treatment group and time was tested using the likelihood ratio test. In the presence of an interaction, the analysis was stratified by treatment group, and a mixed model with occasions nested within individuals was performed separately for each group. These stratified analyses were adjusted in two models. Model 1 was adjusted for age and sex, and Model 2 was, furthermore, adjusted for educational level and marital status. The level of significance used was $p<0.05$. When the interaction term was not significant at 5% but was significant at 10%, a sensitivity analysis was performed by exploring the results of the analysis stratified by intervention group (PBL and control). The statistical analysis was performed in SPSS 27 and Stata 16.0.

Results

Sample

Of the initial 446 invited participants, 289 were excluded. Of the latter, 23 did not fulfil the inclusion criteria, 246 declined to participate, and 20 did not respond to the invitation. The final study group consisted of 157 participants, of whom 79 were assigned to the PBL group and 78 assigned to the control group. At the three-year follow-up there were 5 dropouts, 2 participants (1 from the control group and 1 from the PBL group) had not completed the study for personal reasons, and 3 participants had died (2 from the control group and 1 from the PBL group), leaving n=77 participants (PBL) and n=75 (control group) at the three-year follow up. At the five-year follow-up there were 9 dropouts, 2 participants (1 from the control

For peer review only - http://bmjopen.bmj.com/site/about/guidelines.xhtml
For peer review only

group and 1 from the PBL group) had advanced in their illness, and 7 participants had died (3 from the control group and 4 from the PBL group). There was no statistically significant difference in the dropout rate between treatment groups as tested by chi-squared test (*p*=0.980).

Of the total sample of 157 participants, the mean age was 68.7 years ± 8.5 and the majority were men n = 122, 77.7 % (Table 1). Almost half of the participants lived in sub-urban areas, and the remaining lived in small towns or the countryside. In total, 113 participants were retired and the remaining were in employment. Almost half of the participants had suffered from myocardial infarction, which occurred at a mean of 284 days before the start of the study.

Table 1. Baseline characteristics of the participants in the COR-PRIM study randomized to problem-based learning intervention (PBL) or home-sent patient information (control group).

	Total sample (N=157)	PBL (n=79)	Control (n=78)	p-value
Age year, mean (SD)	68.7 (8.5)	68.5 (9.2)	68.9 (7.7)	0.78[a]
Sex, n (%)				
Male	122 (77.7)	60 (75.9)	62 (79.5)	
Female	35 (22.3)	19 (24.1)	16 (20.5)	0.70[b]
Marital status, n (%)				
Cohabiting	115 (74.2)	60 (75.9)	55 (72.4)	
Living alone	40 (25.8)	19 (24.1)	21 (27.6)	0.71[b]
Education level, n (%)				

For peer review only - http://bmjopen.bmj.com/site/about/guidelines.xhtml

Compulsory	84 (54.2)	46 (58.2)	38 (50.0)	
Upper secondary	31 (20.0)	16 (20.3)	25 (19.7)	
University	38 (24.5)	17 (21.5)	21 (27.6)	0.43[b]
SWE-CES total score, mean (SD)	36.8 (5.3)	36.5 (4.9)	37.1 (5.8)	0.49[a]
GSES total score, mean (SD)	31.30 (5.13)	31.20 (5.44)	31.41 (4.82)	0.80[a]
EQ-VAS, mean (SD)	70.21 (17.9)	68.13 (19.5)	72.44 (15.80)	0.05 [a]
HDL-C, mmol/L, mean (SD)	1.27 (0.44)	1.24 (0.39)	1.30 (0.48)	0.42[a]
BMI, kg/m2, mean (SD)	27.10 (4.39)	27.12 (3.91)	27.07 (4.85)	0.95[a]
Smoking, n (%)	19 (12.1)	9 (11.4)	10 (12.8)	0.81[b]

SWE-CES=Patient empowerment, high score implies stronger empowerment;

GSES=General Self-Efficacy, high score implies high general self-efficacy.

EQ-VAS=health status; HDL-C=high-density lipoprotein cholesterol; BMI=Body Mass Index kg/m2;

a=Independent sample *t-test*

b=Chi-square test

At baseline, the majority had no symptoms of angina pectoris. Less than half of the participants had hyperlipidemia. Additional characteristics are available from a previous study [19]. The distribution for continuous outcomes for the PBL and control groups at the one, three and five-year follow-ups is illustrated with a summary of data including minimum, first quartile, median, third quartile and maximum in boxplots (Figure 2). The results of the statistical analysis of study outcomes over time and by intervention are summarized below (Table 2). Results of the stratified analysis adjusted for age and sex only gave results similar

For peer review only - http://bmjopen.bmj.com/site/about/guidelines.xhtml

to the model also adjusted for educational level and marital status. Therefore, table 2 presents only results adjusted for age, sex, educational level, and marital status.

For peer review only

For peer review only - http://bmjopen.bmj.com/site/about/guidelines.xhtml

Table 2. Differences in self-management and cardiac risk factors between problem-based learning intervention (PBL) vs home-sent information (control group). Stratified analysis is adjusted for age, sex, educational level and marital status. Mixed linear model analyses were performed for (SWE-CES, GSES, EQ-VAS, HDL-C, BMI and Weight) and mixed logistic regression for (smoking).

			PBL group			Control group		
	Outcome	*p*-value	N	Mean (SD) or n (%)	*p*-value	N	Mean (SD) or n (%)	*p*-value
Overall effect of group	SWE-CES	0.558						
Overall effect of time		0.113						
Time x group		0.086[b]						
Baseline		Ref	76	36.54 (4.94)	Ref	72	37.15 (5.81)	Ref
Time 1		0.386	57	37.79 (5.22)	0.119	54	36.57 (5.53)	0.720
Time 3		0.060	56	38.34 (5.76)	0.023	55	37.80 (5.76)	0.834
Time 5		0.025	49	39.39 (5.23)	< 0.001	47	37.21 (5.09)	0.721
Overall effect of group	GSES	0.791						
Overall effect of time		0.312						
Time x group		0.331[c]						
Baseline		Ref	75	31.20 (5.44)	-	71	31.41 (4.82)	-
Time 1		0.201	61	31.07 (4.66)	-	58	31.88 (4.99)	-
Time 3		0.793	57	31.56 (5.26)	-	54	31.67 (5.56)	-
Time 5		0.123	55	32.69 (5.46)	-	52	31.83 (4.70)	-
Overall effect of group	EQ-VAS	0.875						
Overall effect of time		0.209						
Time x group		0.022[a]						
Baseline		Ref	76	68.13 (19.53)	Ref	71	72.44 (15.80)	Ref
Time 1		0.038	59	74.64 (18.05)	0.026	60	75.30 (16.36)	0.501
Time 3		0.256	56	78.27 (14.84)	0.007	56	71.89 (18.33)	0.195
Time 5		0.462	57	77.33 (15.52)	0.031	51	72.06 (17.19)	0.137
Overall effect of group	HDL-C	0.555						
Overall effect of time		0.002						
Time x group		0.016[a]						
Baseline		Ref	74	1.24 (0.39)	Ref	75	1.30 (0.48)	Ref
Time 1		0.075	59	1.37 (0.43)	0.003	60	1.23 (0.36)	0.781
Time 3		< 0.001	47	1.42 (0.55)	< 0.001	44	1.27 (0.37)	0.655
Time 5		0.036	58	1.39 (0.41)	< 0.001	55	1.27 (0.42)	0.459
Overall effect of group	BMI	0.962						

For peer review only

Overall effect of time		0.973						
Time x group		0.061[b]						
Baseline		Ref	65	27.12 (3.91)	Ref	67	27.07 (4.85)	Ref
Time 1		0.951	58	26.83 (3.93)	0.076	56	27.83 (4.97)	0.100
Time 3		0.673	43	27.33 (4.22)	0.440	44	28.04 (4.77)	0.874
Time 5		0.897	53	27.11 (4.43)	0.701	52	27.78 (5.07)	0.862
Overall effect of group	Weight	0.772						
Overall effect of time		0.885						
Time x group		0.047[a]						
Baseline		Ref	74	81.75 (13.61)	Ref	74	81.44 (17.45)	Ref
Time 1		0.739	61	81.77 (13.58)	0.085	61	84.05 (17.19)	0.045
Time 3		0.607	47	83.83 (15.52)	0.788	46	86.40 (18.64)	0.372
Time 5		0.426	56	84.52 (15.16)	0.864	57	86.15 (18.20)	0.408
Overall effect of group	Smoking	0.029						
Overall effect of time		0.428						
Time x group		0.787[c]						
Baseline		Ref	79	7 (8.9)	-	76	8 (10.5)	-
Time 1		0.971	62	4 (6.5)	-	63	9 (14.2)	-
Time 3		0.309	48	5 (10.4)	-	52	9 (17.3)	-
Time 5		0.461	65	3 (4.6)	-	65	7 (10.8)	-

SWE-CES=Patient empowerment; GSES=General Self-Efficacy; EQ-VAS=health status; HDL-C=high-density lipoprotein cholesterol, mmol/L; BMI=Body Mass Index, kg/m2

a=Interaction year x group p<.05. Analysis stratified by groups (PBL) and (Control group)

b=Interaction year x group p<.10. Analysis stratified by group (PBL) and (Control group)

c=Interaction year x group p>.10. Analysis not stratified

Time 1=One year follow-up

Time 3=Three years follow-up

Time 5=Five years follow-up

For peer review only

Findings of the intervention of PBL on primary and secondary outcomes

Patient empowerment

There was a statistically significant change over time of patient SWE-CES (patient empowerment) from baseline to time 5 (p = 0.025) in the total group of participants (Table 2). Additional findings of post hoc sensitivity analysis with interaction test significant at 10% was found for SWE-CES. The interaction between time and group was (p = 0.086). This implies that the analysis of SWE-CES stratified by group showed a significant increase in the PBL group as a part of the sensitivity analysis. SWE-CES increased significantly at time 3 (M = 38.34 ± 5.76, p = 0.023) and time 5 (M = 39.39 ± 5.23, p <0.001) (Table 2) compared to the baseline, independently of positive effects of co-variates low education (p = 0.041); it did not, however, change significantly over time in the control group. Being a woman did, however, negatively effect SWE-CES (p = 0.010).

Self-rated health

We observed a statistically significant change of EQ-VAS (self-rated health status) from baseline to time 1 (p = 0.038). The interaction between time and group was significant at the level of 5% (p = 0.022). Therefore, the analysis was stratified by group. EQ-VAS increased significantly at time 1 (M = 74.64 ± 18.05, p = 0.026), time 3 (M = 78.27 ± 14.84, p = 0.007), and time 5 (M = 77.33 ± 15.52, p = 0.031) in the PBL group compared to baseline, independently of negative effects of co-variates age (p = 0.018), while positive effects of low education increased EQ-VAS by 10 units compared to high education (p = 0.011). No significant changes were observed in the control group at time 1 (M = 75.30 ± 16.36, p = 0.501), time 3 (M = 71.89 ± 18.33, p = 0.195), and time 5 (M = 72.06 ± 17.19, p = 0.137) compared to baseline (Table 2). The co-variate living alone showed a negative effect on EQ-VAS by -7.5 units compared with living together (p = 0.039).

For peer review only - http://bmjopen.bmj.com/site/about/guidelines.xhtml

High density lipoprotein cholesterol

There was a statistically significant change of HDL-C (high density lipoprotein cholesterol) from baseline to time 5 (p = 0.036) (Table 2). The interaction between time and group was significant at alpha level 5% (p = 0.016). Therefore, the analysis was stratified by group. HDL-C increased significantly at time 1 (M = 1.37 mmol/L ± 0.43, p = 0.003), time 3 (M = 1.42 mmol/L ± 0.55, p <0.001), and time 5 (M = 1.39 mmol/L ± 0.41, p <0.001) in the PBL group compared to baseline, independently of the positive effects of co-variates sex (woman) (p = 0.002) and negative effects of low education compared high education (p = 0.035). There was no significant change in the control group at time 1 (M = 1.23 ± 0.36, p = 0.781), time 3 (M = 1.27 ± 0.37, p = 0.655) or time 5 (M = 1.27 ± 0.42, p = 0.459) compared to baseline (Table 2). However, being a woman was positively associated with HDL-C (p = 0.026).

Body Mass Index and weight

There was no statistically significant main effect (time or group) for BMI (Body Mass Index) (Table 2). There was a significant interaction between time and group at the significance level 10% (p = 0.061). Therefore, the analysis was stratified by group. The analysis stratified by group showed a slight decrease from baseline (M = 27.12 ± 3.91) after 1-year (M = 26.83 ± 3.93, p = 0.076), but that did not remain over time. No significant changes over time were observed for the control group (Table 2). For weight, there was a significant interaction between time and group at alpha level of 5% (p = 0.047). Therefore, the analysis was stratified by group. No significant differences were observed between time 1 and 5 in the PBL group compared to baseline. However, the mean weight of patients in the control group at time 1 increased significantly compared to baseline (p = 0.045) (Table 2).

Self-efficacy

For GSES (self-efficacy) there was no significant group effect or change over time (Table 2).

For peer review only - http://bmjopen.bmj.com/site/about/guidelines.xhtml

For peer review only

Smoking

There was no significant interaction between time and intervention ($p = 0.787$), but there was a significant group effect ($p = 0.029$) for smoking, showing that the overall proportion of smokers in the control group was statistically significantly higher than the proportion of smokers in the PBL group (Table 2).

Discussion

We investigated the long-term effects of a one-year PBL intervention in primary care on patient empowerment, self-efficacy, health status and cardiac risk factors in patients with CHD. PBL improved patient empowerment, health status and HDL-C over the 5 years follow-up compared to control group. However, PBL did not result in changes in BMI, weight, smoking or self-efficacy.

We observed significant changes from PBL in patient empowerment and health status over the five-year follow-up compared to the one-year follow-up study [19]. It seems possible that it might take a long time to adapt to longstanding behavioral changes. As many studies in this area often end within a year [9,10,35] our study indicates that long term follow-up is needed to understand the effects on patient behavioural outcomes of interventions using social cognitive theories. Other research shows the need for long-term data on effectiveness of patient education about how to lessen risk factors after CHD [36].

Cardiac rehabilitation reduces risk factors but may also improve health over time [37].
A national prospective cohort study with nearly 4,500 participants showed that cardiac rehabilitation had positive effects on quality of life up to one year after hospital discharge compared to non-participants [38]. In our study, health status improved up to one year, and also after three and five years compared to the control group. PBL allows patients to actively

For peer review only - http://bmjopen.bmj.com/site/about/guidelines.xhtml

decide what is important to discuss in cardiac rehabilitation, suggesting that PBL might promote control over the disease, leading to better health.

We found that HDL-C improved over the five years follow-up. Similar results have been reported in short term follow-up studies on cardiac risk factors using combined education and written information intensive health education sessions and individual support [39,40]. These studies share a focus on education, but we have used PBL in group sessions in a period of one year. PBL activates the participants' problem-solving skills, which is not possible with home-sent patient information. Increased patient empowerment can lead to lifestyle changes as well as decisions to make no changes at all. Improvements in patient empowerment in our study indicate a clinically relevant change in for example HDL-C that also consisted as clinically relevant after 5 years follow-up. Our findings confirm that cardiac rehabilitation focusing on behavioral strategies may be beneficial in maintaining healthy levels of HDL-C.

Multidisciplinary weigh loss behavioral interventions in cardiac rehabilitation may be effective in the short run [41]. We did not observe any changes in BMI between or within groups over time. In contrast, a meta-analysis study involving almost 20,000 participants showed that internet-based education significantly reduced cardiac risk factors regarding blood pressure, blood lipids, weight and physical inactivity, with changes lasting up to one year [35]. We did not include physical activity which could have been valuable to gain a deeper understanding of the non-significant results of BMI and weight over the 5 years follow-up. A cognitive nurse-led intervention designed to improve physical activity by using repeated telephone calls and text messaging consultations improved BMI at a 6-month follow-up compared with controls [42]. Our study was also nurse-led, but the intervention did not include extra consultations, telephone calls or text message for achieving lifestyle goals. Digital aids with continuous support may be helpful when new health promotion activities are being started [43,44].

For peer review only - http://bmjopen.bmj.com/site/about/guidelines.xhtml

The results from the Euroaspire V study reported a high prevalence of persistent smokers after a CHD event [13]. In this study, PBL did not result in a successful lifestyle change in smoking during the follow-up. Even though few participants were smokers in our study, there was a trend, albeit not a significant one, of participants in the control group being smokers more often than those in the PBL group. Higher age, low level of education and living alone were also significantly associated with smoking. As reported from other research, these socio-demographic aspects could be considered in stratifying for cardiac risk factors [45].

We observed no long-term effects of PBL on self-efficacy. This finding is in contrast to Su et al, who reported significant improvements in self-efficacy from an eHealth cardiac rehabilitation intervention [46]. Both studies included cardiac patients and used social cognitive theory methods. However, the inconsistency can probably be explained by the facts that participants in our study were older, several lived alone, and the majority had compulsory education. We accounted for these factors, suggesting that future studies may need to take sociodemographic factors into account in studies designed for cardiac rehabilitation interventions.

The COR-PRIM study has some study limitations and strengths that we would like to address. First, it is a challenge to manage problems arising due to loss of follow-up and deaths while performing a study with long-term follow up design. During a longitudinal study, participants are exposed by several things for example social media, TV campaigns and articles. This affects both control and intervention participants. However, participants in PBL group have learned to appraise patient information as more or less evident. Publicity in various media can influence the attitude towards important secondary preventive factors such as treatment with statins [47]. It is therefore important that the PBL group are supported with an evidence-based approach. Furthermore, participants received a strategy during the one-year intervention to

For peer review only - http://bmjopen.bmj.com/site/about/guidelines.xhtml

reflect on public information presented in for example newspapers and television. They also felt empowered in a new way to discuss treatment and self-care in the group [48].

Secondly, an increased patient empowerment (SWE-CES) in patients may not result in adherence to guidelines. Most of the patients were retired and co-habiting, which may imply that lifestyles were influenced by partners [49]. They were invited to some of the PBL sessions to take part in discussions about their own questions with health care professionals, but we did not follow up the spouses after the intervention. However, the PBL-intervention was performed for one year, and we believe this is a strength, as the education became a part of the patients' lives.

Patients were recruited 6-12 months after the cardiac event on the basis that many continue to smoke, live with hypertension and elevated cholesterol levels about six months after starting the medication [13] which indicate that patients returned to old habits as before the cardiac event. Another clinical observation was that many patients wait for the visit to the cardiologist after discharge from the hospital. However, this is delayed with several months due to heavy workload, leaving the patients to themselves. Thus, we suggest that our intervention fills a gap during the rehabilitation process. A major strength and novelty of this study is that it was performed in primary care after the hospital-based rehabilitation programme. This fact also explains why it was so long time after the event.

Most of the participants in this study were men, living in sub-urban areas and were retired. This should be considered as barriers during implementation of PBL programme in primary care settings. Some of these barriers for example geographic distance and retirement could be addressed by offering a digital intervention. To mitigate the unequal distribution of men and woman, analysis was adjusted for sex. All participants had similar opportunities to undergo traditional cardiac school for one day. Cardiac school may have influenced participants attitude and knowledge and the results in this study. However, the primary outcome in this

For peer review only - http://bmjopen.bmj.com/site/about/guidelines.xhtml

study was patient empowerment, and completion of cardiac school before the intervention might have low influence on patient empowerment. Another aspect is the high number of participants that did not want to be included. This can be partly due to the reluctance to be in a study and having to schedule meetings and follow-up visits. We have seen this recently in another study in a similar patient group [50] in which the lack of time and other commitments (e.g., travel or taking care of grandchildren) were presented as main reasons of non-participation. This has consequences of the generalizability of the study results.

A major strength of our study is the long-term follow-up, which is very much needed in interventions aiming at cardiac preventive behavioral changes. Another strength is that the intervention was led by pedagogical trained specialist nurses who worked in primary care. Patient education led by health care professionals skilled in the chosen educational model improve patients' knowledge, empowerment, and management of the disease [51]. A further advantage is that patients could choose to participate in group education, but few chose to participate, a digital PBL education may therefore be a future option.

Conclusion

One-year PBL intervention had positive effect on patient empowerment, health status and HDL-C compared to the control group but did not result in improvements in other cardiac risk factors or self-efficacy. Co-variates age, sex, education, and marital status emerge both as healthy and cardiac risk factors.

Acknowledgments: We would like to thank study participants and nurses who contributed to the PBL intervention and for the monitoring during the follow-ups.

Contributors: CA, PT, SN, TJ and AKK contributed to the conception or design of the work. AKK contributed to the acquisition of the work and CA, PT, SN, TJ, NK and AKK contributed to analysis or interpretation of data for the work. CA drafted the manuscript and

For peer review only - http://bmjopen.bmj.com/site/about/guidelines.xhtml

AKK, PT, SN, TJ, NK made substantial intellectual contributions to the manuscript. All authors agree to be accountable for all parts of the manuscript and approve the publication of the final manuscript.

Funding: This work was supported by the Swedish Heart and Lung Association project numbers E091/10, E122/11, E083/12, and E103/13 and the Country Council of Region Östergötland, Sweden, project numbers LIO-92281, − 125151, − 27535, − 354951, and LIO-433801.

Competing interests: None declared

Patients consent for publication: Written consent was obtained directly from patients and before study start.

Ethics approval: This study involves human participants and was approved by Regional Ethics Committee of Linköping, Dnr 2010/128-31.

Provenance and peer review: Not commissioned; externally peer reviewed.

Data availability statement: Primary and secondary data will be shared to researcher who provide a methodological proposal with a clear aim. Proposals should be directed to the principal investigator AKK and data access agreement should be completed. All individual deidentified data published in the article are available up on reasonable request. Data will be available 01.01.23 and until 30.06.23. The study protocol is available [17].

For peer review only

References

1. Timmis A, Townsend N, Gale CP, *et al.* European Society of Cardiology: Cardiovascular Disease Statistics 2019. *Eur Heart J* 2020;41:12-85.doi:10.1093/eurheartj/ehz859

2. WHO. Global Health Estimates 2020: Deaths by Cause, Age, Sex, by Country and by Region, 2000-2019. Geneva, World Health Organization; 2020. https://www.who.int/data/gho/data/themes/mortality-and-global-health-estimates (accessed 26 May 2022)

3. Ambrosetti M, Abreu A, Corra U, *et al.* Secondary prevention through comprehensive cardiovascular rehabilitation: From knowledge to implementation. 2020 update. A position paper from the Secondary Prevention and Rehabilitation Section of the European Association of Preventive Cardiology. *Eur J Prev Cardiol* 2020;10.1177/2047487320913379:2047487320913379.doi:10.1177/2047487320913379

4. Novaković M, Novak T, Cuderman TV, *et al.* Exercise capacity improvement after cardiac rehabilitation following myocardial infarction and its association with long-term cardiovascular events. *Eur J Cardiovasc Nurs* 2022;21:76-84.doi:10.1093/eurjcn/zvab015

5. Novaković M, Rajkovič U, Košuta D, *et al.* Effects of Cardiac Rehabilitation and Diet Counselling on Adherence to the Mediterranean Lifestyle in Patients after Myocardial Infarction. *Nutrients* 2022;14:4048.doi:10.3390/nu14194048

6. Sandra M, Maria Miguel R, Ana B, *et al.* Long-term effects of a cardiac rehabilitation program in the control of cardiovascular risk factors. *Rev Port Cardiol* 2013;32:191-199.doi:10.1016/j.repce.2013.03.001

7. Edström Plüss C, Billing E, Held C, *et al.* Long-term effects of an expanded cardiac rehabilitation programme after myocardial infarction or coronary artery bypass surgery: a five-year follow-up of a randomized controlled study. *Clin Rehabil* 2011;25:79-87.doi:10.1177/0269215510376006

For peer review only - http://bmjopen.bmj.com/site/about/guidelines.xhtml

8. Pack QR, Goel K, Squires RW*, et al.* Participation in cardiac rehabilitation and survival after coronary artery bypass graft surgery: A community-based study. *Circulation* 2013;128:590-597-597.doi:10.1161/CIRCULATIONAHA.112.001365

9. Mills M, Loney P, Jamieson E*, et al.* A primary care cardiovascular risk reduction clinic in Canada was more effective and no more expensive than usual on-demand primary care – a randomised controlled trial. *Health SoC Care Community* 2010;18:30-40.doi:10.1111/j.1365-2524.2009.00872.x

10. Storm V, Dorenkamper J, Reinwand DA*, et al.* Effectiveness of a Web-Based Computer-Tailored Multiple-Lifestyle Intervention for People Interested in Reducing their Cardiovascular Risk: A Randomized Controlled Trial. *J Med Internet Res* 2016;18:e78.doi:10.2196/jmir.5147

11. Goyer L, Dufour R, Janelle C*, et al.* Randomized controlled trial on the long-term efficacy of a multifaceted, interdisciplinary lifestyle intervention in reducing cardiovascular risk and improving lifestyle in patients at risk of cardiovascular disease. *J Behavioral Med* 2013;36:212-224.doi:10.1007/s10865-012-9407-3

12. Ferrières J, Lautsch D, Ambegaonkar BM*, et al.* Use of guideline-recommended management in established coronary heart disease in the observational DYSIS II study. *Int J Cardiol* 2018;270:21-27.doi:10.1016/j.ijcard.2018.06.008

13. Kotseva K, De Backer G, De Bacquer D*, et al.* Lifestyle and impact on cardiovascular risk factor control in coronary patients across 27 countries: Results from the European Society of Cardiology ESC-EORP EUROASPIRE V registry. *Eur J Prev Cardiol* 2019;26:824-835.doi:10.1177/2047487318825350

14. Olsen SJ, Schirmer H, Bonaa KH*, et al.* Cardiac rehabilitation after percutaneous coronary intervention: Results from a nationwide survey. *Eur J Cardiovasc Nurs* 2018;17:273-279.doi:10.1177/1474515117737766

For peer review only - http://bmjopen.bmj.com/site/about/guidelines.xhtml

15. Gomez-Velasco DV, Almeda-Valdes P, Martagon AJ*, et al.* Empowerment of patients with type 2 diabetes: current perspectives. *Diabetes Metab Syndr Obes* 2019;12:1311-1321.doi:10.2147/DMSO.S174910

16. Williams B, Pace AE. Problem based learning in chronic disease management: A review of the research. *Patient Educ Couns* 2009;77:14-19.doi:10.1016/j.pec.2009.03.004

17. Karner A, Nilsson S, Jaarsma T*, et al.* The effect of problem-based learning in patient education after an event of CORONARY heart disease--a randomised study in PRIMARY health care: design and methodology of the COR-PRIM study. *BMC Fam Pract* 2012;13:110.doi:10.1186/1471-2296-13-110

18. Lilly CL, Bryant LL, Leary JM*, et al.* Evaluation of the effectiveness of a problem-solving intervention addressing barriers to cardiovascular disease prevention behaviors in 3 underserved populations: Colorado, North Carolina, West Virginia, 2009. *Prev Chronic Dis* 2014;11:E32.doi:10.5888/pcd11.130249

19. Köhler AK, Jaarsma T, Tingström P*, et al.* The effect of problem-based learning after coronary heart disease - a randomised study in primary health care (COR-PRIM). *BMC Cardiovasc Disord* 2020;20:370.doi:10.1186/s12872-020-01647-2

20. Ogmundsdottir Michelsen H, Sjolin I, Schlyter M*, et al.* Cardiac rehabilitation after acute myocardial infarction in Sweden - evaluation of programme characteristics and adherence to European guidelines: The Perfect Cardiac Rehabilitation (Perfect-CR) study. *Eur J Prev Cardiol* 2020;27:18-27.doi:10.1177/2047487319865729

21. Tingström P, Kamwendo K, Göransson A*, et al.* Validation and feasibility of problem-based learning in rehabilitation of patients with coronary artery disease. *Patient Educ Couns* 2002;47:337-345.doi:10.1016/S0738-3991(02)00007-1

For peer review only - http://bmjopen.bmj.com/site/about/guidelines.xhtml

22. Leksell J, Funnell M, Sandberg G, *et al.* Psychometric properties of the Swedish Diabetes Empowerment Scale. *Scand J Caring Sci* 2007;21:247-252.doi:10.1111/j.1471-6712.2007.00463.x

23. Gadermann AM, Zumbo BD, Zeisser C. Ordinal versions of coefficients alpha and theta for likert rating scales. *J Mod Appl Stat Methods* 2007;6:21-29.doi:10.22237/jmasm/1177992180

24. Löve J, Moore Crystal D, Hensing G. Validation of the Swedish translation of the general self-efficacy scale. *Qual Life Res* 2012;21:1249-1253.doi:

25. Tanimura C, Aoto H, Kobayashi N, *et al.* Effects of a Self-efficacy Theory-Based Training Program for Peers of Patients with Type 2 Diabetes. *Yonago Acta Med* 2020;63:282-293.doi:10.33160/yam.2020.11.006

26. Tan CCL, Hwang SW, Zhang N, *et al.* Effect of a Diabetes Self-Efficacy Enhancing Program on Older Adults With Type 2 Diabetes: A Randomized Controlled Trial. *Clin Nurs Res* 2020;29:293-303.doi:10.1177/1054773818792480

27. Tang CY, Blackstock FC, O'Halloran PD, *et al.* A Standard Form of Cardiac Rehabilitation Can Improve Self-Reported Positive Behavior Changes in the Short- to Medium-Term. *J Cardiopulm Rehabil* 2019;39:175-180.doi:10.1097/HCR.0000000000000371

28. Borzou SR, Amiri S, Salavati M, *et al.* Effects of the First Phase of Cardiac Rehabilitation Training on Self-Efficacy among Patients Undergoing Coronary Artery Bypass Graft Surgery. *J Tehran Univ Heart Center* 2018;13:126-131.doi:

29. Madueño-Caro AJ, Pardos-Lafarga M, Muñoz-Ayllón M, *et al.* Cardiac rehabilitation in primary care. Impact of an intervention on perceived self-efficacy. *J Functional Morphol* 2019;410.3390/jfmk4020032

For peer review only - http://bmjopen.bmj.com/site/about/guidelines.xhtml

30. Lenz ER, Shortridge-Baggett LM. Self efficacy in nursing. research and measurement perspectives: Springer Pub.; 2002.

31. EuroQol Research Foundation. EQ-5D-Y User Guide. https://euroqol.org/publications/user-guides/

32. Cheng LJ, Tan RL, Luo N. Measurement Properties of the EQ VAS Around the Globe: A Systematic Review and Meta-Regression Analysis. *Value Health* 2021;24:1223-1233.doi:10.1016/j.jval.2021.02.003

33. Gurka MJ, Filipp SL, Musani SK*, et al.* Use of BMI as the marker of adiposity in a metabolic syndrome severity score: Derivation and validation in predicting long-term disease outcomes. *Metab Clin Exp* 2018;83:68-74.doi:10.1016/j.metabol.2018.01.015

34. Gibson RS. Principles of nutritional assessment. New York: Oxford University Press; 2005.

35. Beishuizen CR, Stephan BC, van Gool WA*, et al.* Web-Based Interventions Targeting Cardiovascular Risk Factors in Middle-Aged and Older People: A Systematic Review and Meta-Analysis. *J Med Internet Res* 2016;18:e55.doi:10.2196/jmir.5218

36. Dürsch M, Melamed RJ, Tillmann A*, et al.* Evaluating the efficacy of an education and treatment program for patients with coronary heart disease - A randomized controlled trial. *Dtsch Arztebl* 2014;111:802-808-808.doi:10.3238/arztebl.2014.0802

37. Hansen VB, Maindal HT. Cardiac rehabilitation with a nurse case manager (GoHeart) across local and regional health authorities improves risk factors, self-care and psychosocial outcomes. A one-year follow-up study. *JRSM Cardiovasc Dis* 2014;3:1-11.doi:10.1177/2048004014555922

38. Hurdus B, Munyombwe T, Dondo TB*, et al.* Association of cardiac rehabilitation and health-related quality of life following acute myocardial infarction. *Heart (British Cardiac Society)* 2020;106:1726-1731.doi:10.1136/heartjnl-2020-316920

For peer review only - http://bmjopen.bmj.com/site/about/guidelines.xhtml

39. Dehghani A, Kumar Bhasin S, Dwivedi S, *et al.* Influence of Comprehensive Life Style Intervention in Patients of CHD. *Glob J Health Sci* 2015;7:6-16.doi:10.5539/gjhs.v7n7p6

40. Salas-Salvadó J, Díaz-López A, Basora J, *et al.* Effect of a lifestyle intervention program with energy-restricted Mediterranean diet and exercise on weight loss and cardiovascular risk factors: One-year results of the PREDIMED-Plus trial. *Diabetes Care* 2019;42:777-788.doi:10.2337/dc18-0836

41. Gallagher R, Kirkness A, Armari E, *et al.* Weight management issues and strategies for people with high cardiovascular risk undertaking an Australian weight loss program: a focus group study. *Nurs Health Sci* 2012;14:18-24.doi:10.1111/j.1442-2018.2011.00651.x

42. Alsaleh E, Windle R, Blake H. Behavioural intervention to increase physical activity in adults with coronary heart disease in Jordan. *BMC Public Health* 2016;16:643.doi:10.1186/s12889-016-3313-5

43. Karyotaki E, Ebert DD, Donkin L, *et al.* Do guided internet-based interventions result in clinically relevant changes for patients with depression? An individual participant data meta-analysis. *Clin Psychol Rev* 2018;63:80-92.doi:10.1016/j.cpr.2018.06.007

44. Johansson P, Westas M, Andersson G, *et al.* An Internet-Based Cognitive Behavioral Therapy Program Adapted to Patients With Cardiovascular Disease and Depression: Randomized Controlled Trial. *JMIR Ment Health* 2019;6:e14648.doi:10.2196/14648

45. Safford MM, Reshetnyak E, Sterling MR, *et al.* Number of Social Determinants of Health and Fatal and Nonfatal Incident Coronary Heart Disease in the REGARDS Study. *Circulation* 2021;143:244-253.doi:10.1161/circulationaha.120.048026

46. Su JJ, Yu DS. Effects of a nurse-led eHealth cardiac rehabilitation programme on health outcomes of patients with coronary heart disease: A randomised controlled trial. *Int J Nurs Stud* 2021;122:104040.doi:10.1016/j.ijnurstu.2021.104040

For peer review only - http://bmjopen.bmj.com/site/about/guidelines.xhtml

47. Nielsen SF, Nordestgaard BG. Negative statin-related news stories decrease statin persistence and increase myocardial infarction and cardiovascular mortality: a nationwide prospective cohort study. *Eur Heart J* 2016;37:908-916.doi:10.1093/eurheartj/ehv641

48. Hjelmfors L, Abrandt Dahlgren M, Kärner A*, et al.* Problem-based learning used in the context of cardiac rehabilitation: different scenes and different roles. *Australia*, *2014*: 36, p.218-232. Taylor & Francis.

49. Köhler AK, Nilsson S, Jaarsma T*, et al.* Health beliefs about lifestyle habits differ between patients and spouses 1 year after a cardiac event - a qualitative analysis based on the Health Belief Model. *Scand J Caring Sci* 2017;31:332-341.doi:10.1111/scs.12351

50. Poli A, Kelfve S, Klompstra L*, et al.* Prediction of (Non)Participation of Older People in Digital Health Research: Exergame Intervention Study. *J Med Internet Res* 2020;22:e17884.doi:10.2196/17884

51. Horder M, Andersen S. Patient education–a health technology assessment. *Copenhagen: Danish Centre for Health Technology Assessment NBoH* 2009

Figure Legend:

Figure 1. Flowchart on study participation.

Figure 2. Distribution of outcome variables in PBL and control groups at baseline 1, 3 and 5 year follow-up.

Supplemental Material 1

For peer review only - http://bmjopen.bmj.com/site/about/guidelines.xhtml

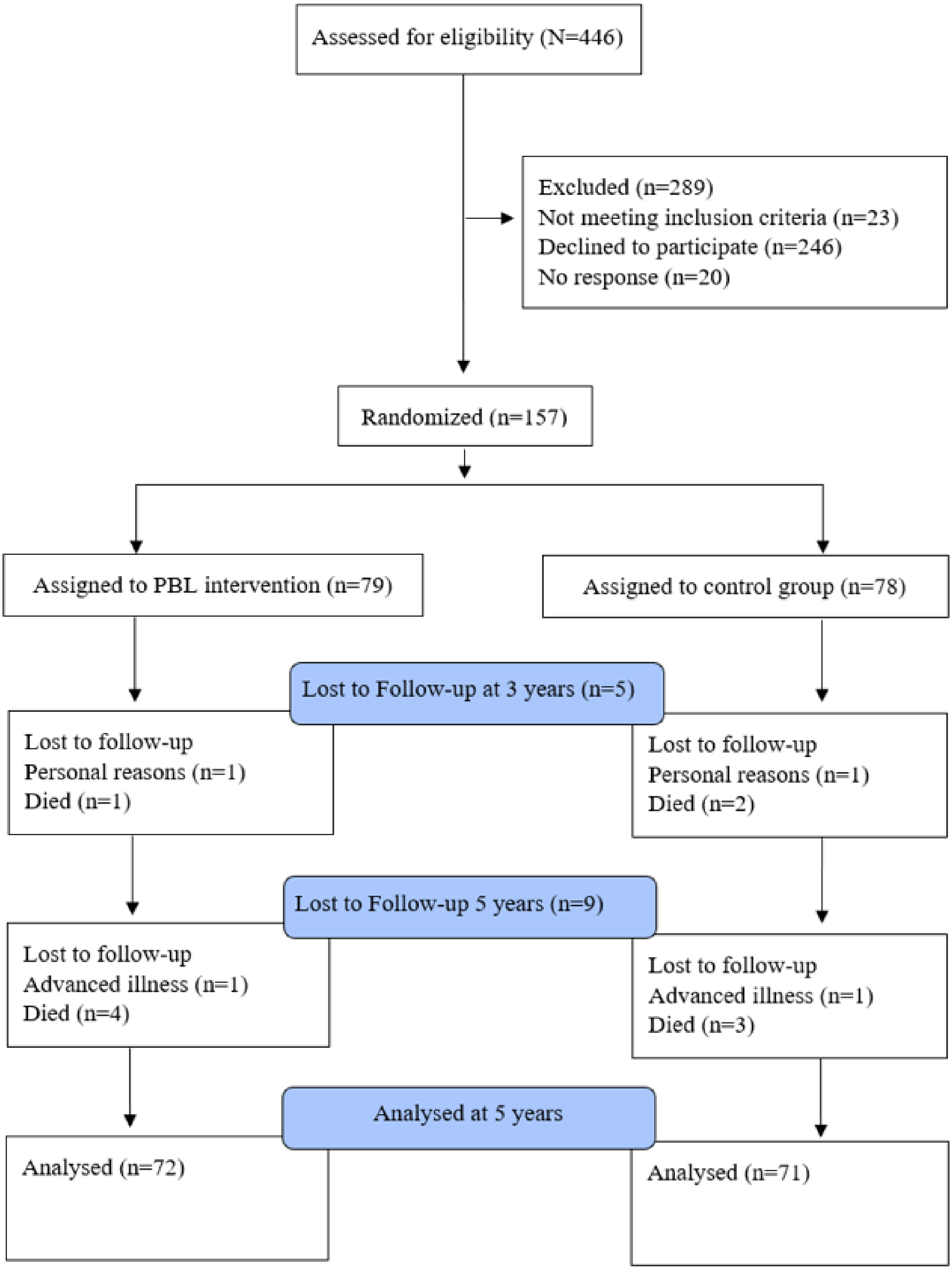

Figure 1. Flowchart on study participation

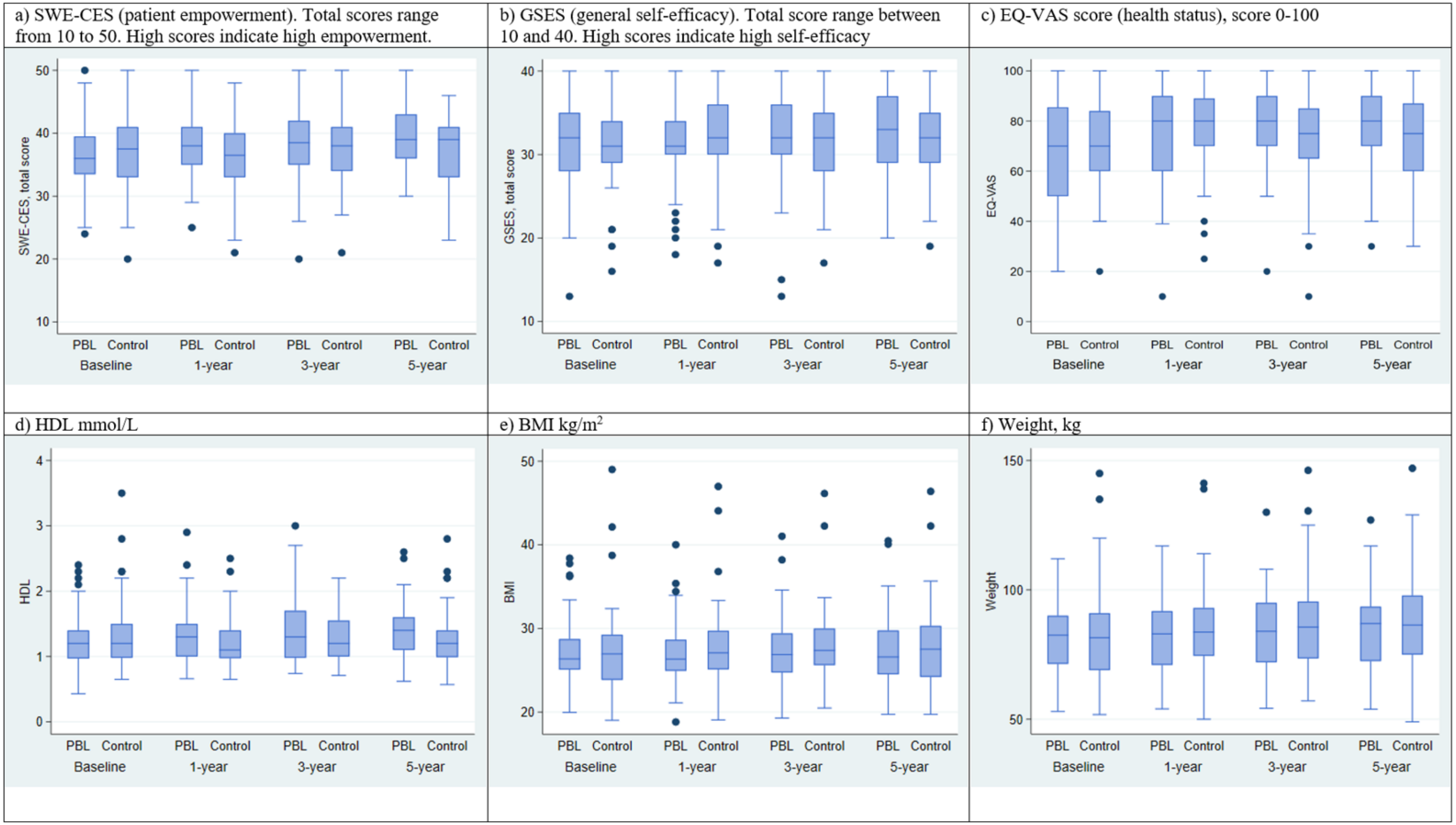

Figure 2. Distribution of outcome variables in PBL and control groups at baseline 1, 3 and 5 year follow-up.

Supplemental material 1 *BMJ open*

Title of the article:
Does problem-based learning improve patient empowerment and cardiac risk factors in patients with coronary heart disease in a Swedish primary care setting? A long-term prospective, randomized, parallel single randomized trial (COR-PRIM).

Authors: Christina Andreae, Pia Tingström, Staffan Nilsson, Tiny Jaarsma, Nadine Karlsson and Anita Kärner Köhler.

Supplement 1. Description of the PBL intervention

The PBL intervention consisted of group learning sessions in primary care led by district nurses who were advanced trained in PBL for two days by the research group. Two of the authors (AKK and PT) supported and tutored the district nurses every month in their development as PBL tutors. Tutoring groups consisted of 6-9 patients and participated in 13 learning sessions (2 hours) over one year. The sessions were every week for the first month, the two next months included two sessions/month, and the next five sessions were at 16, 20, 26, 39, 52 weeks after the start.

A problem-solving process (a PBL-model) supported the patient's formulation of mutual questions and learning goals and individual self-care goals, which they wrote and reflected on in an individual study guide. Triggers e.g., texts including real life scenarios or images were used to start the learning process about CHD, symptoms such as: chest pain, shortness of breath, swollen legs and signs of high blood pressure, cholesterol levels, lifestyle changes, in diet, physical activity, medicines, mental outcomes e.g., depressive mood, anxiety and fears, job life and stress, sex life. Importantly, the patients chose in which order the different scenarios were chosen to work with. This was a way to empower them and make the learning relevant to their situation. The district nurses supported and challenged the patients to select evidence-based literature. When the patients could not solve the questions, resource persons e.g., a general practitioner, physiotherapist were invited. These meetings were in between the group meetings and interested relatives were welcome and did attend.

Patients randomized to the control group received with the same interval as the PBL group, home-sent evidence-based patient information about self-care reflecting a cognitive intervention but did not participate in critical or reflective learning. Group effects were assessed after 11 sessions over one year.

For peer review only

CONSORT 2010 checklist of information to include when reporting a randomised trial*

Section/Topic	Item No	Checklist item	Reported on page No
**Title and abstract**			
	1a	Identification as a randomised trial in the title	1
	1b	Structured summary of trial design, methods, results, and conclusions (for specific guidance see CONSORT for abstracts)	2,3
**Introduction**			
Background and objectives	2a	Scientific background and explanation of rationale	3-5
	2b	Specific objectives or hypotheses	5
**Methods**			
Trial design	3a	Description of trial design (such as parallel, factorial) including allocation ratio	6-7
	3b	Important changes to methods after trial commencement (such as eligibility criteria), with reasons	NA
Participants	4a	Eligibility criteria for participants	6
	4b	Settings and locations where the data were collected	6
Interventions	5	The interventions for each group with sufficient details to allow replication, including how and when they were actually administered	7+ Supplement material +Figure 1
Outcomes	6a	Completely defined pre-specified primary and secondary outcome measures, including how and when they were assessed	7-8 and page 6
	6b	Any changes to trial outcomes after the trial commenced, with reasons	NA
Sample size	7a	How sample size was determined	7
	7b	When applicable, explanation of any interim analyses and stopping guidelines	NA
Randomisation:			
Sequence generation	8a	Method used to generate the random allocation sequence	6-7
	8b	Type of randomisation; details of any restriction (such as blocking and block size)	7
Allocation concealment mechanism	9	Mechanism used to implement the random allocation sequence (such as sequentially numbered containers), describing any steps taken to conceal the sequence until interventions were assigned	7
Implementation	10	Who generated the random allocation sequence, who enrolled participants, and who assigned participants to	7

For peer review only - http://bmjopen.bmj.com/site/about/guidelines.xhtml

		interventions	
Blinding	11a	If done, who was blinded after assignment to interventions (for example, participants, care providers, those assessing outcomes) and how	7
	11b	If relevant, description of the similarity of interventions	NA
Statistical methods	12a	Statistical methods used to compare groups for primary and secondary outcomes	9,10
	12b	Methods for additional analyses, such as subgroup analyses and adjusted analyses	10
**Results**			
Participant flow (a diagram is strongly recommended)	13a	For each group, the numbers of participants who were randomly assigned, received intended treatment, and were analysed for the primary outcome	Figure 1
	13b	For each group, losses and exclusions after randomisation, together with reasons	Figure 1
Recruitment	14a	Dates defining the periods of recruitment and follow-up	6
	14b	Why the trial ended or was stopped	NA
Baseline data	15	A table showing baseline demographic and clinical characteristics for each group	Table 1
Numbers analysed	16	For each group, number of participants (denominator) included in each analysis and whether the analysis was by original assigned groups	Table 2
Outcomes and estimation	17a	For each primary and secondary outcome, results for each group, and the estimated effect size and its precision (such as 95% confidence interval)	NA
	17b	For binary outcomes, presentation of both absolute and relative effect sizes is recommended	NA
Ancillary analyses	18	Results of any other analyses performed, including subgroup analyses and adjusted analyses, distinguishing pre-specified from exploratory	16-18 + Table 2
Harms	19	All important harms or unintended effects in each group (for specific guidance see CONSORT for harms)	NA
**Discussion**			
Limitations	20	Trial limitations, addressing sources of potential bias, imprecision, and, if relevant, multiplicity of analyses	20-22
Generalisability	21	Generalisability (external validity, applicability) of the trial findings	22
Interpretation	22	Interpretation consistent with results, balancing benefits and harms, and considering other relevant evidence	20-22
**Other information**			
Registration	23	Registration number and name of trial registry	6
Protocol	24	Where the full trial protocol can be accessed, if available	Ref 17
Funding	25	Sources of funding and other support (such as supply of drugs), role of funders	23

For peer review only - http://bmjopen.bmj.com/site/about/guidelines.xhtml

*We strongly recommend reading this statement in conjunction with the CONSORT 2010 Explanation and Elaboration for important clarifications on all the items. If relevant, we also recommend reading CONSORT extensions for cluster randomised trials, non-inferiority and equivalence trials, non-pharmacological treatments, herbal interventions, and pragmatic trials. Additional extensions are forthcoming: for those and for up to date references relevant to this checklist, see www.consort-statement.org.

For peer review only

For peer review only - http://bmjopen.bmj.com/site/about/guidelines.xhtml

BMJ Open

Does problem-based learning improve patient empowerment and cardiac risk factors in patients with coronary heart disease in a Swedish primary care setting? A long-term prospective, randomized, parallel single randomized trial (COR-PRIM).

Journal:	*BMJ Open*
Manuscript ID	bmjopen-2022-065230.R2
Article Type:	Original research
Date Submitted by the Author:	21-Dec-2022
Complete List of Authors:	Andreae, Christina; Linkoping University Department of Health Medicine and Caring Sciences,<br>Tingström, Pia; Linkoping University Department of Health Medicine and Caring Sciences<br>Nilsson, Staffan; Linkoping University Department of Health Medicine and Caring Sciences<br>Jaarsma, Tiny; Linkoping University Department of Health Medicine and Caring Sciences<br>Karlsson, Nadine; Linkoping University Department of Health Medicine and Caring Sciences<br>Köhler, Anita Kärner; Linkoping University Department of Health Medicine and Caring Sciences
**Primary Subject Heading**:	Cardiovascular medicine
Secondary Subject Heading:	Medical education and training
Keywords:	Coronary heart disease < CARDIOLOGY, PRIMARY CARE, EDUCATION & TRAINING (see Medical Education & Training)

SCHOLARONE™
Manuscripts

I, the Submitting Author has the right to grant and does grant on behalf of all authors of the Work (as defined in the below author licence), an exclusive licence and/or a non-exclusive licence for contributions from authors who are: i) UK Crown employees; ii) where BMJ has agreed a CC-BY licence shall apply, and/or iii) in accordance with the terms applicable for US Federal Government officers or employees acting as part of their official duties; on a worldwide, perpetual, irrevocable, royalty-free basis to BMJ Publishing Group Ltd ("BMJ") its licensees and where the relevant Journal is co-owned by BMJ to the co-owners of the Journal, to publish the Work in this journal and any other BMJ products and to exploit all rights, as set out in our licence.

The Submitting Author accepts and understands that any supply made under these terms is made by BMJ to the Submitting Author unless you are acting as an employee on behalf of your employer or a postgraduate student of an affiliated institution which is paying any applicable article publishing charge ("APC") for Open Access articles. Where the Submitting Author wishes to make the Work available on an Open Access basis (and intends to pay the relevant APC), the terms of reuse of such Open Access shall be governed by a Creative Commons licence – details of these licences and which Creative Commons licence will apply to this Work are set out in our licence referred to above.

Other than as permitted in any relevant BMJ Author's Self Archiving Policies, I confirm this Work has not been accepted for publication elsewhere, is not being considered for publication elsewhere and does not duplicate material already published. I confirm all authors consent to publication of this Work and authorise the granting of this licence.

For peer review only

Does problem-based learning improve patient empowerment and cardiac risk factors in patients with coronary heart disease in a Swedish primary care setting? A long-term prospective, randomized, parallel single randomized trial (COR-PRIM).

Christina Andreae[1,2], Pia Tingström[1], Staffan Nilsson[3], Tiny Jaarsma[1,4] Nadine Karlsson[1] and Anita Kärner Köhler[1]

1. Department of Health, Medicine and Caring Sciences, Linköping University, Linköping, Sweden.

2. Centre for Clinical Research Sörmland, Uppsala University, Eskilstuna, Sweden.

3. Primary Health Care Center Vikbolandet, and Department of Health, Medicine and Caring Sciences, Linköping University, Linköping, Sweden.

4. Julius Center, University Medical Center Utrecht, Utrecht, the Netherlands.

Corresponding author: Christina Andreae, PhD, RN, Department of Medicine, Region Sörmland, 63188 Eskilstuna, Sweden

Email: christina.andreae@liu.se

Tel: +4616 10 44 07

For peer review only

For peer review only - http://bmjopen.bmj.com/site/about/guidelines.xhtml

Abstract

Objectives: To investigate long-term effects of a one-year problem-based learning (PBL) on self-management and cardiac risk factors in patients with coronary heart disease (CHD).

Design: A prospective, randomized, parallel single centre trial.

Settings: Primary care settings in Sweden.

Participants: 157 patients with stable CHD completed the study. Subjects with reading and writing impairments, mental illness or expected survival less than one year were excluded.

Intervention: Participants were randomized and assigned to receive either PBL (intervention) or home-sent patient information (control group). In this study participants were followed-up at baseline 1, 3 and 5 years.

Primary and secondary outcomes: Primary outcome was patient empowerment (SWE-CES) and secondary outcomes general self-efficacy (GSES), self-rated health status (EQ-VAS), high-density lipoprotein cholesterol (HDL-C), Body Mass Index (BMI), weight and smoking. Outcomes were adjusted for sociodemographic factors.

Results: The PBL intervention group resulted in a significant improved change in SWE-CES over the five-year period (Mean [M], 39.39; 95% confidence interval [CI], 37.88-40.89) compared to the baseline (M, 36.54; CI, 35.40-37.66). PBL intervention group increased HDL-C level (M, 1,39; CI, 1.28-1.50) compared to baseline (M, 1,24; CI, 1.15-1.33) and for EQ-VAS (M, 77.33; CI, 73.21-81.45) compared to baseline (M, 68.13; CI, 63.66-72.59) while these outcomes remained unchanged in the control group. There were no significant differences in BMI, weight, or scores on GSES, neither between nor within groups over time. The overall proportion of smokers was significantly higher in the control group than in the experimental group.

Conclusion: One-year PBL intervention had positive effect on patient empowerment, health status and HDL-C at a five-year follow-up compared to the control group. PBL education

For peer review only - http://bmjopen.bmj.com/site/about/guidelines.xhtml

aiming to improve patient empowerment in cardiac rehabilitation should account for sociodemographic factors.

Keywords: Coronary disease, empowerment, heart disease risk factors, long-term, primary health care, problem-based learning, randomized controlled trial.

Trial registration: The study was registered at ClinicalTrial.gov with number NCT01462799.

For peer review only

Strengths and limitations of this study:

- The majority of participants remained in the study over the 5-years follow-up.
- Baseline measurement was well balanced for both groups regarding sociodemographic and study variables.
- The PBL intervention was held by a small group of specialist nurses who were advanced trained in PBL.
- Participants were most retired, were men and lived in sub-urban areas which need to be considered in the implementation of PBL in cardiac primary care settings.

Introduction

Nearly 126 million people globally suffer from coronary heart disease (CHD), the leading cause of death worldwide [1,2]. Important treatment goals include slowing down the underlying atherosclerosis process by targeting hypertension, high cholesterol, diabetes, overweight, tobacco use, alcohol consumption, physical inactivity, and poor diet [3]. Cardiac rehabilitation in post-myocardial infarction improve lifestyle habits such as physical activity and dietary intake, and has also shown positive effects on blood lipids, blood pressure and smoking [4-6]. In

For peer review only - http://bmjopen.bmj.com/site/about/guidelines.xhtml

a small randomized trial, cardiac rehabilitation programme for patients with myocardial infarction or who had undergone coronary artery by-pass graft surgery (CABG), focusing on stress management, physical exercise and dietary intake, reduced morbidity and hospitalization for CHD [7]. Attendance in cardiac rehabilitation programme has long-term effects on survival among patients who have undergone CABG with reduced 10-year all-cause mortality [8]. Cardiac rehabilitation enables patients to make lifestyle changes that are important for maintaining health. Cardiac rehabilitation consists of multidisciplinary interventions where patients are offered tailored education and counselling with the goals of improving health behavior for sustainable secondary prevention [3]. Several studies have investigated the effect of interventions such as nurse telephone follow-up or group education, and described the effects on illness perception, self-efficacy, behaviour change and cardiac risk factors [9-11]. The effects of several programme on cardiac risk factors or behaviour were found from 3 months up to 2 years. Patients' attendance of cardiac rehabilitation appears to be low, and risk factors remain or even deteriorate from the first to second cardiac event [12-14]. For example, persistent smoking is common after discharge from hospital, and intentions to quit smoking in the near future remain low. This regards also overweight and physical activity after a cardiac event, many patients do not lose weight and nearly half of patients perform no physical activity and do not make changes to their physical status [13]. Thus, primary care has great challenges motivating patients to achieve healthy lifestyles.

Empowering patients to take control over the disease, and making them aware of factors that affect illness, have positively impacted on health outcomes in chronic diseases [15]. Problem-based learning (PBL) is a method that empowers patients to become aware of how to reduce risk factors in chronic diseases [16]. PBL is a cognitive educational model that promotes self-learning where critical and reflective thinking are important components of learning outcomes and enhanced self-management. PBL is process-oriented, meaning that learning skills are

For peer review only - http://bmjopen.bmj.com/site/about/guidelines.xhtml

developed through active, creative, and cognitive processes. Previous knowledge is placed in the light of real situations where knowledge is developed in a context together with others, all led by a trained PBL tutor [17]. A focus group intervention with problem solving intervention in a cardiac population showed that an eight-week focus group sessions resulted in significant improvements of stress management, dietary intake and physical activity [18]. The first results from a one-year follow-up in a coronary heart disease in primary care study (COR-PRIM) showed no differences in patient empowerment or self-efficacy between PBL intervention and patients who received home-sent patient information (controls). However, significant differences in secondary outcomes – i.e., body mass index, body weight and high-density lipoprotein cholesterol (HDL-C) – were found between the groups, with the PBL group performing favourably [19].

Although cardiac rehabilitation in Sweden has recently been assessed to be high quality [20] participation remains low. Only a few interventions incorporating a holistic view of health compromising psychological- and social aspects of health behaviors have been performed on the group level. PBL may empower self-management. The present study is elaborated in accordance with the COR-PRIM study basic aim, which was to discover whether PBL provided in primary health care for one year has long-term effects on patient empowerment and self-care, assessed at baseline and, at one, three, and five years after randomization. In this five year- and final assessment we wanted to identify if the findings in the 1-year follow-up remained or changed [19]. By this performance, this article is examining the sustainability of the effects by PBL, which to our knowledge has not been performed before.

Thus, the purpose of this study was to investigate the long-term effects on patient empowerment, self-efficacy, health status and cardiac risk factors in patients with CHD of a one-year PBL intervention in primary care, compared to home-sent patient information.

For peer review only

For peer review only - http://bmjopen.bmj.com/site/about/guidelines.xhtml

Methods

Design, sample and procedure

The COR-PRIM study was a prospective, randomized, parallel, and single centre trial designed to investigate whether one year of a PBL programme had long-term effects on patient empowerment, self-efficacy and cardiac risk factors [17]. The study protocol is registered at ClinicalTrial.gov, with the registration number NCT01462799. Patients diagnosed with CHD verified by percutaneous coronary intervention (PCI) or coronary artery by-pass surgery (CABG) and CABG+PCI or myocardial infarction within 6-12 months prior to the intervention and who had previously completed cardiac rehabilitation were eligible for the study. Additional criteria were that patients were stable in their heart disease, pharmacologically optimized in the last month before inclusion and, if applicable had completed cardiac school in the clinic. Patients with impaired ability to communicate and read in Swedish, verified psychiatric illness or short expected survival (of less than one year) were excluded [17]. The recruitment started in November 2011 and assessments of primary and secondary outcomes were collected until November 2019. Nurses at outpatient cardiac clinics identified eligible patients through medical records. These patients were then invited to participate in the study at a regular nurse-led cardiac follow-up visit. Patients received oral and written study information from a research assistant, and written consent was obtained from those interested in participating. Supplemental Material 1.

The study randomization and intervention

In COR-PRIM, a total of 157 patients were randomized and assigned to either problem-based learning intervention (PBL) or home-sent patient information (control group). The randomization procedure was based on block randomization with 18 study numbers based on a minimum of 12-18 study participants. Block randomization was performed using sealed and

For peer review only - http://bmjopen.bmj.com/site/about/guidelines.xhtml

unmarked envelopes. An assistant, blinded to the randomization and located outside the research setting, randomly allocated patients to either the PBL group (experimental) or home-sent patient information (control group) at a 1:1 ratio [17]. Nurses who performed the PBL intervention were not blinded, as the intervention was based on problem-based learning, which is different from regular care. The PBL intervention consisted of group learning sessions in primary care led by nurses who had advanced training in leading problem-based learning. Tutoring groups consisted of 6-9 participants and participated in 13 sessions (2 hours per meeting) over one year. This was based on the multiple of areas to discuss for lifestyles changes in CHD. For further information about the PBL intervention see Supplemental Material 2. Lifestyles changes require work over time and to be feasible in the patient's life, a long training is required. The PBL model has been validated as feasible in clinical settings [21]. Patients randomized to the control group received home-sent patient information reflecting a cognitive intervention, but did not participate in critical or reflective learning. Group effects were assessed after 11 sessions over one year [17].

Patient and public involvement

There were no patient or public involvement in the study.

Sample size

Sample size was calculated based on the primary outcome variable i.e., patient empowerment. The expected means for the Swedish Coronary Empowerment Scale (SWE-CES) were 30 and 36 for the control group and the PBL group respectively. With an estimated significance level of α=5% and a power of 1-β=80%, the sample size resulted in a minimum of 63 participants in each group [17]. Analyses conducted for the five-year follow-up period was n=72 (PBL) and n=71 (control group) (Figure 1).

Primary and secondary outcome measures

Patient empowerment was the study's primary outcome and was assessed at baseline, 1, 3 and

For peer review only - http://bmjopen.bmj.com/site/about/guidelines.xhtml

5 years (i.e., baseline, time 1, time 3 and time 5 respectively). Secondary outcomes included general self-efficacy while five secondary outcomes were added post hoc and these were health status, high-density lipoprotein cholesterol, body mass index, weight and smoking and were assessed at baseline, 1, 3 and 5 years.

Measurement

Sociodemographic variables and covariates

Sociodemographic variables consisting of age, sex, education level, marital status, place of residence, employment and smoking were determined by self-reported questionnaires. The covariates consisted of age, sex, education level and marital status.

Self-reported measures

The Swedish coronary empowerment scale (SWE-CES) measures how patients achieve goals, overcome barriers for goals achievement, and use the strategies necessary to make self-care choices. The strategies include dimensions of coping in managing disease, stress and dissatisfaction and also readiness to make health changes. The instrument contains 10 items that have 5 response alternatives ranged by Likert type options from 1 to 5. The total score ranges between 10 and 50, where high scores imply high level of patient empowerment. Four subscales measure different aspects of patient empowerment [22]. In this study, the total scores of the SWE-CES scale were used. Internal consistency reliability indicated acceptable values with Cronbach's alfa 0.751 [23].

Self-efficacy was measured by using the general self-efficacy scale (GSES). The instrument consists of 10 items that measure a person's belief in their own ability to implement behavioral changes in order to reduce risk factors for unhealthy lifestyles. Items are rated on a four-point Likert type scale ranging from 1 (not at all true) to 4 (exactly true). The total score ranges from 10 to 40, where a high total score indicates higher general self-efficacy [24]. Self-efficacy has been used to evaluate interventions to strengthen the self-care capacity of patients

For peer review only - http://bmjopen.bmj.com/site/about/guidelines.xhtml

with various chronic diseases such as diabetes [25,26] and patients with CHD [27-29]. It has been translated and psychometrically evaluated among a general Swedish population [30]. In this study, internal consistency reliability indicated acceptable values with Cronbach's alfa 0.914.

Health status

Self-rated health was assessed using EuroQol (EQ-VAS). The scale represents patients' overall health status and consists of a score between 0 and 100, where 0 indicates worst health and 100 the best health [31]. EQ-VAS has shown acceptable construct validity across populations [32].

Cardiac risk factors

High density lipoprotein cholesterol (HDL-C) was assessed using blood samples collected and analysed according to normal clinical routines. Anthropometric measurements of body size included length, weight, and Body Mass Index (BMI). Length and weight were measured in light cloths and with shoes removed. BMI is a widely used clinical measure and is recommended as indicator for defining obesity in adults. It is, furthermore, a reliable anthropometric measure in predicting metabolic syndromes [1,33]. BMI was calculated by dividing weight by the square of height in meters using the formula (weight (kg) / height (m^2)) [34]. Tobacco use was self-reported at each measure point.

Statistical analysis

The descriptive statistics included mean standard deviation (SD) or frequencies percent (%). Independent sample t-tests or Chi-square tests were used to analyses differences in sociodemographic and data characteristics between groups. Because the occasions (baseline to five-year) are nested within individuals, we employed a two-level mixed linear model with occasions ("time" at level 1) that are nested within individuals (level 2) to analyze continuous outcomes. The random part of the variance components model is individuals. The normality of continuous study outcomes was assessed using Shapiro-Wilk test. If the outcomes were

For peer review only - http://bmjopen.bmj.com/site/about/guidelines.xhtml

non-normally distributed, they were log transformed prior to the analysis. Smoking is a binary outcomes and was analysed with a logistic mixed model. The statistical analysis of each study outcome was performed using a mixed linear model with treatment group, age and sex as fixed variables, with occasions nested within individuals, and an interaction of time by treatment group. The interaction (combined effect) between treatment group and time was tested using the likelihood ratio test. In the presence of an interaction, the analysis was stratified by treatment group, and a mixed model with occasions nested within individuals was performed separately for each group. These stratified analyses were adjusted in two models. Model 1 was adjusted for age and sex, and Model 2 was, furthermore, adjusted for educational level and marital status. The level of significance used was $p<0.05$. When the interaction term was not significant at 5% but was significant at 10%, a sensitivity analysis was performed by exploring the results of the analysis stratified by intervention group (PBL and control). The statistical analysis was performed in SPSS 27 and Stata 16.0.

For peer review only

Results

Sample

Of the initial 446 invited participants, 289 were excluded. Of the latter, 23 did not fulfil the inclusion criteria, 246 declined to participate, and 20 did not respond to the invitation. The final study group consisted of 157 participants, of whom 79 were assigned to the PBL group and 78 assigned to the control group. At the three-year follow-up there were 5 dropouts, 2 participants (1 from the control group and 1 from the PBL group) had not completed the study for personal reasons, and 3 participants had died (2 from the control group and 1 from the PBL group), leaving n=77 participants (PBL) and n=75 (control group) at the three-year follow up. At the five-year follow-up there were 9 dropouts, 2 participants (1 from the control

For peer review only - http://bmjopen.bmj.com/site/about/guidelines.xhtml

group and 1 from the PBL group) had advanced in their illness, and 7 participants had died (3 from the control group and 4 from the PBL group). There was no statistically significant difference in the dropout rate between treatment groups as tested by chi-squared test (p=0.980).

Of the total sample of 157 participants, the mean age was 68.7 years ± 8.5 and the majority were men n = 122, 77.7 % (Table 1). Almost half of the participants lived in sub-urban areas, and the remaining lived in small towns or the countryside. In total, 113 participants were retired and the remaining were in employment. Almost half of the participants had suffered from myocardial infarction, which occurred at a mean of 284 days before the start of the study.

Table 1. Baseline characteristics of the participants in the COR-PRIM study randomized to problem-based learning intervention (PBL) or home-sent patient information (control group).

	Total sample (N=157)	PBL (n=79)	Control (n=78)	p-value
Age year, mean (SD)	68.7 (8.5)	68.5 (9.2)	68.9 (7.7)	0.78[a]
Sex, n (%)				
Male	122 (77.7)	60 (75.9)	62 (79.5)	
Female	35 (22.3)	19 (24.1)	16 (20.5)	0.70[b]
Marital status, n (%)				
Cohabiting	115 (74.2)	60 (75.9)	55 (72.4)	
Living alone	40 (25.8)	19 (24.1)	21 (27.6)	0.71[b]
Education level, n (%)				

For peer review only - http://bmjopen.bmj.com/site/about/guidelines.xhtml

Compulsory	84 (54.2)	46 (58.2)	38 (50.0)	
Upper secondary	31 (20.0)	16 (20.3)	25 (19.7)	
University	38 (24.5)	17 (21.5)	21 (27.6)	0.43[b]
SWE-CES total score, mean (SD)	36.8 (5.3)	36.5 (4.9)	37.1 (5.8)	0.49[a]
GSES total score, mean (SD)	31.30 (5.13)	31.20 (5.44)	31.41 (4.82)	0.80[a]
EQ-VAS, mean (SD)	70.21 (17.9)	68.13 (19.5)	72.44 (15.80)	0.05 [a]
HDL-C, mmol/L, mean (SD)	1.27 (0.44)	1.24 (0.39)	1.30 (0.48)	0.42[a]
BMI, kg/m2, mean (SD)	27.10 (4.39)	27.12 (3.91)	27.07 (4.85)	0.95[a]
Smoking, n (%)	19 (12.1)	9 (11.4)	10 (12.8)	0.81[b]

SWE-CES=Patient empowerment, high score implies stronger empowerment;

GSES=General Self-Efficacy, high score implies high general self-efficacy.

EQ-VAS=health status; HDL-C=high-density lipoprotein cholesterol; BMI=Body Mass Index kg/m2;

a=Independent sample *t-test*

b=Chi-square test

At baseline, the majority had no symptoms of angina pectoris. Less than half of the participants had hyperlipidemia. Additional characteristics are available from a previous study [19]. The distribution for continuous outcomes for the PBL and control groups at the one, three and five-year follow-ups is illustrated with a summary of data including minimum, first quartile, median, third quartile and maximum in boxplots (Figure 2). The results of the statistical analysis of study outcomes over time and by intervention are summarized below (Table 2). Results of the stratified analysis adjusted for age and sex only gave results similar

For peer review only - http://bmjopen.bmj.com/site/about/guidelines.xhtml

to the model also adjusted for educational level and marital status. Therefore, table 2 presents only results adjusted for age, sex, educational level, and marital status.

For peer review only

For peer review only - http://bmjopen.bmj.com/site/about/guidelines.xhtml

Table 2. Differences in self-management and cardiac risk factors between problem-based learning intervention (PBL) vs home-sent information (control group). Stratified analysis is adjusted for age, sex, educational level and marital status. Mixed linear model analyses were performed for (SWE-CES, GSES, EQ-VAS, HDL-C, BMI and Weight) and mixed logistic regression for (smoking).

				PBL group			Control group	
	Outcome	*p*-value	N	Mean (SD) or n (%)	*p*-value	N	Mean (SD) or n (%)	*p*-value
Overall effect of group	SWE-CES	0.558						
Overall effect of time		0.113						
Time x group		0.086[b]						
Baseline		Ref	76	36.54 (4.94)	Ref	72	37.15 (5.81)	Ref
Time 1		0.386	57	37.79 (5.22)	0.119	54	36.57 (5.53)	0.720
Time 3		0.060	56	38.34 (5.76)	0.023	55	37.80 (5.76)	0.834
Time 5		0.025	49	39.39 (5.23)	< 0.001	47	37.21 (5.09)	0.721
Overall effect of group	GSES	0.791						
Overall effect of time		0.312						
Time x group		0.331[c]						
Baseline		Ref	75	31.20 (5.44)	-	71	31.41 (4.82)	-
Time 1		0.201	61	31.07 (4.66)	-	58	31.88 (4.99)	-
Time 3		0.793	57	31.56 (5.26)	-	54	31.67 (5.56)	-
Time 5		0.123	55	32.69 (5.46)	-	52	31.83 (4.70)	-
Overall effect of group	EQ-VAS	0.875						
Overall effect of time		0.209						
Time x group		0.022[a]						
Baseline		Ref	76	68.13 (19.53)	Ref	71	72.44 (15.80)	Ref
Time 1		0.038	59	74.64 (18.05)	0.026	60	75.30 (16.36)	0.501
Time 3		0.256	56	78.27 (14.84)	0.007	56	71.89 (18.33)	0.195
Time 5		0.462	57	77.33 (15.52)	0.031	51	72.06 (17.19)	0.137
Overall effect of group	HDL-C	0.555						
Overall effect of time		0.002						
Time x group		0.016[a]						
Baseline		Ref	74	1.24 (0.39)	Ref	75	1.30 (0.48)	Ref
Time 1		0.075	59	1.37 (0.43)	0.003	60	1.23 (0.36)	0.781
Time 3		< 0.001	47	1.42 (0.55)	< 0.001	44	1.27 (0.37)	0.655
Time 5		0.036	58	1.39 (0.41)	< 0.001	55	1.27 (0.42)	0.459
Overall effect of group	BMI	0.962						

For peer review only - http://bmjopen.bmj.com/site/about/guidelines.xhtml

Overall effect of time		0.973						
Time x group		0.061[b]						
Baseline		Ref	65	27.12 (3.91)	Ref	67	27.07 (4.85)	Ref
Time 1		0.951	58	26.83 (3.93)	0.076	56	27.83 (4.97)	0.100
Time 3		0.673	43	27.33 (4.22)	0.440	44	28.04 (4.77)	0.874
Time 5		0.897	53	27.11 (4.43)	0.701	52	27.78 (5.07)	0.862
Overall effect of group	Weight	0.772						
Overall effect of time		0.885						
Time x group		0.047[a]						
Baseline		Ref	74	81.75 (13.61)	Ref	74	81.44 (17.45)	Ref
Time 1		0.739	61	81.77 (13.58)	0.085	61	84.05 (17.19)	0.045
Time 3		0.607	47	83.83 (15.52)	0.788	46	86.40 (18.64)	0.372
Time 5		0.426	56	84.52 (15.16)	0.864	57	86.15 (18.20)	0.408
Overall effect of group	Smoking	0.029						
Overall effect of time		0.428						
Time x group		0.787[c]						
Baseline		Ref	79	7 (8.9)	-	76	8 (10.5)	-
Time 1		0.971	62	4 (6.5)	-	63	9 (14.2)	-
Time 3		0.309	48	5 (10.4)	-	52	9 (17.3)	-
Time 5		0.461	65	3 (4.6)	-	65	7 (10.8)	-

SWE-CES=Patient empowerment; GSES=General Self-Efficacy; EQ-VAS=health status; HDL-C=high-density lipoprotein cholesterol, mmol/L; BMI=Body Mass Index, kg/m2

a=Interaction year x group *p*<.05. Analysis stratified by groups (PBL) and (Control group)

b=Interaction year x group *p*<.10. Analysis stratified by group (PBL) and (Control group)

c=Interaction year x group *p*>.10. Analysis not stratified

Time 1=One year follow-up

Time 3=Three years follow-up

Time 5=Five years follow-up

For peer review only - http://bmjopen.bmj.com/site/about/guidelines.xhtml

For peer review only

Findings of the intervention of PBL on primary and secondary outcomes

Patient empowerment

There was a statistically significant change over time of patient SWE-CES (patient empowerment) from baseline to time 5 (p = 0.025) in the total group of participants (Table 2). Additional findings of post hoc sensitivity analysis with interaction test significant at 10% was found for SWE-CES. The interaction between time and group was (p = 0.086). This implies that the analysis of SWE-CES stratified by group showed a significant increase in the PBL group as a part of the sensitivity analysis. SWE-CES increased significantly at time 3 (M = 38.34 ± 5.76, p = 0.023) and time 5 (M = 39.39 ± 5.23, p <0.001) (Table 2) compared to the baseline, independently of positive effects of co-variates low education (p = 0.041); it did not, however, change significantly over time in the control group. Being a woman did, however, negatively effect SWE-CES (p = 0.010).

Self-rated health

We observed a statistically significant change of EQ-VAS (self-rated health status) from baseline to time 1 (p = 0.038). The interaction between time and group was significant at the level of 5% (p = 0.022). Therefore, the analysis was stratified by group. EQ-VAS increased significantly at time 1 (M = 74.64 ± 18.05, p = 0.026), time 3 (M = 78.27 ± 14.84, p = 0.007), and time 5 (M = 77.33 ± 15.52, p = 0.031) in the PBL group compared to baseline, independently of negative effects of co-variates age (p = 0.018), while positive effects of low education increased EQ-VAS by 10 units compared to high education (p = 0.011). No significant changes were observed in the control group at time 1 (M = 75.30 ± 16.36, p = 0.501), time 3 (M = 71.89 ± 18.33, p = 0.195), and time 5 (M = 72.06 ± 17.19, p = 0.137) compared to baseline (Table 2). The co-variate living alone showed a negative effect on EQ-VAS by -7.5 units compared with living together (p = 0.039).

For peer review only - http://bmjopen.bmj.com/site/about/guidelines.xhtml

High density lipoprotein cholesterol

There was a statistically significant change of HDL-C (high density lipoprotein cholesterol) from baseline to time 5 (p = 0.036) (Table 2). The interaction between time and group was significant at alpha level 5% (p = 0.016). Therefore, the analysis was stratified by group. HDL-C increased significantly at time 1 (M = 1.37 mmol/L ± 0.43, p = 0.003), time 3 (M = 1.42 mmol/L ± 0.55, p <0.001), and time 5 (M = 1.39 mmol/L ± 0.41, p <0.001) in the PBL group compared to baseline, independently of the positive effects of co-variates sex (woman) (p = 0.002) and negative effects of low education compared high education (p = 0.035). There was no significant change in the control group at time 1 (M = 1.23 ± 0.36, p = 0.781), time 3 (M = 1.27 ± 0.37, p = 0.655) or time 5 (M = 1.27 ± 0.42, p = 0.459) compared to baseline (Table 2). However, being a woman was positively associated with HDL-C (p = 0.026).

Body Mass Index and weight

There was no statistically significant main effect (time or group) for BMI (Body Mass Index) (Table 2). There was a significant interaction between time and group at the significance level 10% (p = 0.061). Therefore, the analysis was stratified by group. The analysis stratified by group showed a slight decrease from baseline (M = 27.12 ± 3.91) after 1-year (M = 26.83 ± 3.93, p = 0.076), but that did not remain over time. No significant changes over time were observed for the control group (Table 2). For weight, there was a significant interaction between time and group at alpha level of 5% (p = 0.047). Therefore, the analysis was stratified by group. No significant differences were observed between time 1 and 5 in the PBL group compared to baseline. However, the mean weight of patients in the control group at time 1 increased significantly compared to baseline (p = 0.045) (Table 2).

Self-efficacy

For GSES (self-efficacy) there was no significant group effect or change over time (Table 2).

For peer review only - http://bmjopen.bmj.com/site/about/guidelines.xhtml

Smoking

There was no significant interaction between time and intervention ($p = 0.787$), but there was a significant group effect ($p = 0.029$) for smoking, showing that the overall proportion of smokers in the control group was statistically significantly higher than the proportion of smokers in the PBL group (Table 2).

Discussion

We investigated the long-term effects of a one-year PBL intervention in primary care on patient empowerment, self-efficacy, health status and cardiac risk factors in patients with CHD. PBL improved patient empowerment, health status and HDL-C over the 5 years follow-up compared to control group. However, PBL did not result in changes in BMI, weight, smoking or self-efficacy.

We observed significant changes from PBL in patient empowerment and health status over the five-year follow-up compared to the one-year follow-up study [19]. It seems possible that it might take a long time to adapt to longstanding behavioral changes. As many studies in this area often end within a year [9,10,35] our study indicates that long term follow-up is needed to understand the effects on patient behavioural outcomes of interventions using social cognitive theories. Other research shows the need for long-term data on effectiveness of patient education about how to lessen risk factors after CHD [36].

Cardiac rehabilitation reduces risk factors but may also improve health over time [37].
A national prospective cohort study with nearly 4,500 participants showed that cardiac rehabilitation had positive effects on quality of life up to one year after hospital discharge compared to non-participants [38]. In our study, health status improved up to one year, and also after three and five years compared to the control group. PBL allows patients to actively

For peer review only - http://bmjopen.bmj.com/site/about/guidelines.xhtml

decide what is important to discuss in cardiac rehabilitation, suggesting that PBL might promote control over the disease, leading to better health.

We found that HDL-C improved over the five years follow-up. Similar results have been reported in short term follow-up studies on cardiac risk factors using combined education and written information intensive health education sessions and individual support [39,40]. These studies share a focus on education, but we have used PBL in group sessions in a period of one year. PBL activates the participants' problem-solving skills, which is not possible with home-sent patient information. Increased patient empowerment can lead to lifestyle changes as well as decisions to make no changes at all. Improvements in patient empowerment in our study indicate a clinically relevant change in for example HDL-C that also consisted as clinically relevant after 5 years follow-up. Our findings confirm that cardiac rehabilitation focusing on behavioral strategies may be beneficial in maintaining healthy levels of HDL-C.

Multidisciplinary weigh loss behavioral interventions in cardiac rehabilitation may be effective in the short run [41]. We did not observe any changes in BMI between or within groups over time. In contrast, a meta-analysis study involving almost 20,000 participants showed that internet-based education significantly reduced cardiac risk factors regarding blood pressure, blood lipids, weight and physical inactivity, with changes lasting up to one year [35]. We did not include physical activity which could have been valuable to gain a deeper understanding of the non-significant results of BMI and weight over the 5 years follow-up. A cognitive nurse-led intervention designed to improve physical activity by using repeated telephone calls and text messaging consultations improved BMI at a 6-month follow-up compared with controls [42]. Our study was also nurse-led, but the intervention did not include extra consultations, telephone calls or text message for achieving lifestyle goals. Digital aids with continuous support may be helpful when new health promotion activities are being started [43,44].

For peer review only - http://bmjopen.bmj.com/site/about/guidelines.xhtml

The results from the Euroaspire V study reported a high prevalence of persistent smokers after a CHD event [13]. In this study, PBL did not result in a successful lifestyle change in smoking during the follow-up. Even though few participants were smokers in our study, there was a trend, albeit not a significant one, of participants in the control group being smokers more often than those in the PBL group. Higher age, low level of education and living alone were also significantly associated with smoking. As reported from other research, these socio-demographic aspects could be considered in stratifying for cardiac risk factors [45].

We observed no long-term effects of PBL on self-efficacy. This finding is in contrast to Su et al, who reported significant improvements in self-efficacy from an eHealth cardiac rehabilitation intervention [46]. Both studies included cardiac patients and used social cognitive theory methods. However, the inconsistency can probably be explained by the facts that participants in our study were older, several lived alone, and the majority had compulsory education. We accounted for these factors, suggesting that future studies may need to take sociodemographic factors into account in studies designed for cardiac rehabilitation interventions.

The COR-PRIM study has some study limitations and strengths that we would like to address. We aimed to include a representative sample of patients with CHD. To enable this, the study sample was based on rigorous inclusion criteria. Participants with CHD that was verified by coronary intervention with PCI, CABG, or previous myocardial infarction. Participants were stable in their CHD and were optimally pharmacologically treated in the last month before inclusion. If applicable, patients should have completed cardiac school at the hospital. With these inclusion criteria, we consider that our sample is representative of a population being diagnosed and treated for CHD. However, the majority in this study were retired and therefore the results need to be interpreted with caution according to a younger population with CHD.

For peer review only - http://bmjopen.bmj.com/site/about/guidelines.xhtml

Most of the participants in this study were men and the majority were living in sub-urban areas. This should be considered as limitation to the generalizability of the results of the study. To mitigate the unequal distribution of men and woman, analysis was adjusted for sex.

Another aspect is the high number of participants that did not want to be included. This can be partly due to the reluctance to be in a study and having to schedule meetings and follow-up visits. We have seen this recently in another study in a similar patient group [47] in which the lack of time and other commitments (e.g., travel or taking care of grandchildren) were presented as reasons of non-participation. This may have consequences of the generalizability of the study results.

Moreover, the poor uptake of the PBL programme could be due to the program design involving 13 sessions for two hours over one year. This may be regarded as challenging to manage especially if patients reside far from the hospital and not having economic compensation, which was a reason for non-participation in a similar PBL programme including 10 sessions. Of 800 screened patients with rheumatoid arthritis nearly 600 patients did not participate and stated for example that they did not want to even if they fit the inclusion criterions [48]. However, patients abandon patient education even if there are few patient education sessions. This was found in the context of diabetes education for three days. Only 24% of the patients attended the education [49]. One way to improve barriers to the uptake of the PBL programme is to offer a digital PBL programme. Digital programme enables people to participate despite living in sub-urban areas who do not have practical or economic resources to travel for a PBL programme. The advantage of digital PBL programme is also that selected parts of the programme could be included as pre-recorded modules. This could make the programme more flexible and accessible to a broader group of patients with CHD, for example for those of employable age. We believe that PBL as a pedagogy, closely offered in a digital way to the patients may be a future option.

For peer review only - http://bmjopen.bmj.com/site/about/guidelines.xhtml

All participants had similar opportunities to undergo traditional cardiac school for one day. Cardiac school may have influenced participants attitude and knowledge and the results in this study. However, the primary outcome in this study was patient empowerment, and completion of cardiac school before the intervention might have low influence on patient empowerment.

It is a challenge to manage problems arising due to loss of follow-up and deaths while performing a study with long-term follow up design. During a longitudinal study, participants are exposed by several things for example social media, TV campaigns and articles. This affects both control and intervention participants. However, participants in PBL group have learned to appraise patient information as more or less evident. Publicity in various media can influence the attitude towards important secondary preventive factors such as treatment with statins [50]. It is therefore important that the PBL group are supported with an evidence-based approach. Furthermore, participants received a strategy during the one-year intervention to reflect on public information presented in for example newspapers and television. They also felt empowered in a new way to discuss treatment and self-care in the group [51].

An increased patient empowerment (SWE-CES) in patients may not result in adherence to guidelines. Most of the patients were retired and co-habiting, which may imply that lifestyles were influenced by partners [52]. They were invited to some of the PBL sessions to take part in discussions about their own questions with health care professionals, but we did not follow up the spouses after the intervention. However, the PBL-intervention was performed for one year, and we believe this is a strength, as the education became a part of the patients' lives.

Patients were recruited 6-12 months after the cardiac event on the basis that many continue to smoke, live with hypertension and elevated cholesterol levels about six months after starting the medication [13] which indicate that patients returned to old habits as before the cardiac event. Another clinical observation was that many patients wait for the visit to the cardiologist after discharge from the hospital. However, this is delayed with several months due to heavy

For peer review only - http://bmjopen.bmj.com/site/about/guidelines.xhtml

workload, leaving the patients to themselves. Thus, we suggest that our intervention fills a gap during the rehabilitation process. A major strength and novelty of this study is that it was performed in primary care after the hospital-based rehabilitation programme. This fact also explains why it was so long time after the event.

A major strength of our study is the long-term follow-up, which is very much needed in interventions aiming at cardiac preventive behavioral changes. Another strength is that the intervention was led by pedagogical trained specialist nurses who worked in primary care. Patient education led by health care professionals skilled in the chosen educational model improve patients' knowledge, empowerment, and management of the disease [53]. A further advantage is that patients could choose to participate in group education, but few chose to participate, a digital PBL education may therefore be a future option.

Conclusion

One-year PBL intervention had positive effect on patient empowerment, health status and HDL-C compared to the control group but did not result in improvements in other cardiac risk factors or self-efficacy. Co-variates age, sex, education, and marital status emerge both as healthy and cardiac risk factors.

Acknowledgments: We would like to thank study participants and nurses who contributed to the PBL intervention and for the monitoring during the follow-ups.

Contributors: CA, PT, SN, TJ and AKK contributed to the conception or design of the work. AKK contributed to the acquisition of the work and CA, PT, SN, TJ, NK and AKK contributed to analysis or interpretation of data for the work. CA drafted the manuscript and AKK, PT, SN, TJ, NK made substantial intellectual contributions to the manuscript. All authors agree to be accountable for all parts of the manuscript and approve the publication of the final manuscript.

For peer review only - http://bmjopen.bmj.com/site/about/guidelines.xhtml

Funding: This work was supported by the Swedish Heart and Lung Association project numbers E091/10, E122/11, E083/12, and E103/13 and the Country Council of Region Östergötland, Sweden, project numbers LIO-92281, − 125151, − 27535, − 354951, and LIO-433801.

Competing interests: None declared

Patients consent for publication: Written consent was obtained directly from patients and before study start.

Ethics approval: This study involves human participants and was approved by Regional Ethics Committee of Linköping, Dnr 2010/128-31.

Provenance and peer review: Not commissioned; externally peer reviewed.

Data availability statement: Primary and secondary data will be shared to researcher who provide a methodological proposal with a clear aim. Proposals should be directed to the principal investigator AKK and data access agreement should be completed. All individual deidentified data published in the article are available up on reasonable request. Data will be available 01.01.23 and until 30.06.23. The study protocol is available [17].

For peer review only

References

1. Timmis A, Townsend N, Gale CP, *et al.* European Society of Cardiology: Cardiovascular Disease Statistics 2019. *Eur Heart J* 2020;41:12-85.doi:10.1093/eurheartj/ehz859

2. WHO. Global Health Estimates 2020: Deaths by Cause, Age, Sex, by Country and by Region, 2000-2019. Geneva, World Health Organization; 2020. https://www.who.int/data/gho/data/themes/mortality-and-global-health-estimates (accessed 26 May 2022)

3. Ambrosetti M, Abreu A, Corra U, *et al.* Secondary prevention through comprehensive cardiovascular rehabilitation: From knowledge to implementation. 2020 update. A position paper from the Secondary Prevention and Rehabilitation Section of the European Association of Preventive Cardiology. *Eur J Prev Cardiol* 2020;10.1177/2047487320913379:2047487320913379.doi:10.1177/2047487320913379

4. Novaković M, Novak T, Cuderman TV, *et al.* Exercise capacity improvement after cardiac rehabilitation following myocardial infarction and its association with long-term cardiovascular events. *Eur J Cardiovasc Nurs* 2022;21:76-84.doi:10.1093/eurjcn/zvab015

5. Novaković M, Rajkovič U, Košuta D, *et al.* Effects of Cardiac Rehabilitation and Diet Counselling on Adherence to the Mediterranean Lifestyle in Patients after Myocardial Infarction. *Nutrients* 2022;14:4048.doi:10.3390/nu14194048

6. Sandra M, Maria Miguel R, Ana B, *et al.* Long-term effects of a cardiac rehabilitation program in the control of cardiovascular risk factors. *Rev Port Cardiol* 2013;32:191-199.doi:10.1016/j.repce.2013.03.001

7. Edström Plüss C, Billing E, Held C, *et al.* Long-term effects of an expanded cardiac rehabilitation programme after myocardial infarction or coronary artery bypass surgery: a five-year follow-up of a randomized controlled study. *Clin Rehabil* 2011;25:79-87.doi:10.1177/0269215510376006

For peer review only - http://bmjopen.bmj.com/site/about/guidelines.xhtml

8. Pack QR, Goel K, Squires RW*, et al.* Participation in cardiac rehabilitation and survival after coronary artery bypass graft surgery: A community-based study. *Circulation* 2013;128:590-597-597.doi:10.1161/CIRCULATIONAHA.112.001365

9. Mills M, Loney P, Jamieson E*, et al.* A primary care cardiovascular risk reduction clinic in Canada was more effective and no more expensive than usual on-demand primary care – a randomised controlled trial. *Health SoC Care Community* 2010;18:30-40.doi:10.1111/j.1365-2524.2009.00872.x

10. Storm V, Dorenkamper J, Reinwand DA*, et al.* Effectiveness of a Web-Based Computer-Tailored Multiple-Lifestyle Intervention for People Interested in Reducing their Cardiovascular Risk: A Randomized Controlled Trial. *J Med Internet Res* 2016;18:e78.doi:10.2196/jmir.5147

11. Goyer L, Dufour R, Janelle C*, et al.* Randomized controlled trial on the long-term efficacy of a multifaceted, interdisciplinary lifestyle intervention in reducing cardiovascular risk and improving lifestyle in patients at risk of cardiovascular disease. *J Behavioral Med* 2013;36:212-224.doi:10.1007/s10865-012-9407-3

12. Ferrières J, Lautsch D, Ambegaonkar BM*, et al.* Use of guideline-recommended management in established coronary heart disease in the observational DYSIS II study. *Int J Cardiol* 2018;270:21-27.doi:10.1016/j.ijcard.2018.06.008

13. Kotseva K, De Backer G, De Bacquer D*, et al.* Lifestyle and impact on cardiovascular risk factor control in coronary patients across 27 countries: Results from the European Society of Cardiology ESC-EORP EUROASPIRE V registry. *Eur J Prev Cardiol* 2019;26:824-835.doi:10.1177/2047487318825350

14. Olsen SJ, Schirmer H, Bonaa KH*, et al.* Cardiac rehabilitation after percutaneous coronary intervention: Results from a nationwide survey. *Eur J Cardiovasc Nurs* 2018;17:273-279.doi:10.1177/1474515117737766

For peer review only - http://bmjopen.bmj.com/site/about/guidelines.xhtml

15. Gomez-Velasco DV, Almeda-Valdes P, Martagon AJ, *et al.* Empowerment of patients with type 2 diabetes: current perspectives. *Diabetes Metab Syndr Obes* 2019;12:1311-1321.doi:10.2147/DMSO.S174910

16. Williams B, Pace AE. Problem based learning in chronic disease management: A review of the research. *Patient Educ Couns* 2009;77:14-19.doi:10.1016/j.pec.2009.03.004

17. Karner A, Nilsson S, Jaarsma T, *et al.* The effect of problem-based learning in patient education after an event of CORONARY heart disease--a randomised study in PRIMARY health care: design and methodology of the COR-PRIM study. *BMC Fam Pract* 2012;13:110.doi:10.1186/1471-2296-13-110

18. Lilly CL, Bryant LL, Leary JM, *et al.* Evaluation of the effectiveness of a problem-solving intervention addressing barriers to cardiovascular disease prevention behaviors in 3 underserved populations: Colorado, North Carolina, West Virginia, 2009. *Prev Chronic Dis* 2014;11:E32.doi:10.5888/pcd11.130249

19. Köhler AK, Jaarsma T, Tingström P, *et al.* The effect of problem-based learning after coronary heart disease - a randomised study in primary health care (COR-PRIM). *BMC Cardiovasc Disord* 2020;20:370.doi:10.1186/s12872-020-01647-2

20. Ogmundsdottir Michelsen H, Sjolin I, Schlyter M, *et al.* Cardiac rehabilitation after acute myocardial infarction in Sweden - evaluation of programme characteristics and adherence to European guidelines: The Perfect Cardiac Rehabilitation (Perfect-CR) study. *Eur J Prev Cardiol* 2020;27:18-27.doi:10.1177/2047487319865729

21. Tingström P, Kamwendo K, Göransson A, *et al.* Validation and feasibility of problem-based learning in rehabilitation of patients with coronary artery disease. *Patient Educ Couns* 2002;47:337-345.doi:10.1016/S0738-3991(02)00007-1

For peer review only - http://bmjopen.bmj.com/site/about/guidelines.xhtml

22. Leksell J, Funnell M, Sandberg G*, et al.* Psychometric properties of the Swedish Diabetes Empowerment Scale. *Scand J Caring Sci* 2007;21:247-252.doi:10.1111/j.1471-6712.2007.00463.x

23. Gadermann AM, Zumbo BD, Zeisser C. Ordinal versions of coefficients alpha and theta for likert rating scales. *J Mod Appl Stat Methods* 2007;6:21-29.doi:10.22237/jmasm/1177992180

24. Löve J, Moore Crystal D, Hensing G. Validation of the Swedish translation of the general self-efficacy scale. *Qual Life Res* 2012;21:1249-1253.doi:

25. Tanimura C, Aoto H, Kobayashi N*, et al.* Effects of a Self-efficacy Theory-Based Training Program for Peers of Patients with Type 2 Diabetes. *Yonago Acta Med* 2020;63:282-293.doi:10.33160/yam.2020.11.006

26. Tan CCL, Hwang SW, Zhang N*, et al.* Effect of a Diabetes Self-Efficacy Enhancing Program on Older Adults With Type 2 Diabetes: A Randomized Controlled Trial. *Clin Nurs Res* 2020;29:293-303.doi:10.1177/1054773818792480

27. Tang CY, Blackstock FC, O'Halloran PD*, et al.* A Standard Form of Cardiac Rehabilitation Can Improve Self-Reported Positive Behavior Changes in the Short- to Medium-Term. *J of Cardiopulm Rehabil* 2019;39:175-180.doi:10.1097/HCR.0000000000000371

28. Borzou SR, Amiri S, Salavati M*, et al.* Effects of the First Phase of Cardiac Rehabilitation Training on Self-Efficacy among Patients Undergoing Coronary Artery Bypass Graft Surgery. *J Tehran Univ Heart Center* 2018;13:126-131.doi:

29. Madueño-Caro AJ, Pardos-Lafarga M, Muñoz-Ayllón M*, et al.* Cardiac rehabilitation in primary care. Impact of an intervention on perceived self-efficacy. *J Functional Morphol* 2019;410.3390/jfmk4020032

For peer review only

30. Lenz ER, Shortridge-Baggett LM. Self efficacy in nursing. research and measurement perspectives: Springer Pub.; 2002.

31. EuroQol Research Foundation. EQ-5D-Y User Guide. https://euroqol.org/publications/user-guides/

32. Cheng LJ, Tan RL, Luo N. Measurement Properties of the EQ VAS Around the Globe: A Systematic Review and Meta-Regression Analysis. *Value Health* 2021;24:1223-1233.doi:10.1016/j.jval.2021.02.003

33. Gurka MJ, Filipp SL, Musani SK*, et al.* Use of BMI as the marker of adiposity in a metabolic syndrome severity score: Derivation and validation in predicting long-term disease outcomes. *Metab Clin Exp* 2018;83:68-74.doi:10.1016/j.metabol.2018.01.015

34. Gibson RS. Principles of nutritional assessment. New York: Oxford University Press; 2005.

35. Beishuizen CR, Stephan BC, van Gool WA*, et al.* Web-Based Interventions Targeting Cardiovascular Risk Factors in Middle-Aged and Older People: A Systematic Review and Meta-Analysis. *J Med Internet Res* 2016;18:e55.doi:10.2196/jmir.5218

36. Dürsch M, Melamed RJ, Tillmann A*, et al.* Evaluating the efficacy of an education and treatment program for patients with coronary heart disease - A randomized controlled trial. *Dtsch Arztebl* 2014;111:802-808-808.doi:10.3238/arztebl.2014.0802

37. Hansen VB, Maindal HT. Cardiac rehabilitation with a nurse case manager (GoHeart) across local and regional health authorities improves risk factors, self-care and psychosocial outcomes. A one-year follow-up study. *JRSM Cardiovasc Dis* 2014;3:1-11.doi:10.1177/2048004014555922

38. Hurdus B, Munyombwe T, Dondo TB*, et al.* Association of cardiac rehabilitation and health-related quality of life following acute myocardial infarction. *Heart (British Cardiac Society)* 2020;106:1726-1731.doi:10.1136/heartjnl-2020-316920

For peer review only

39. Dehghani A, Kumar Bhasin S, Dwivedi S, *et al.* Influence of Comprehensive Life Style Intervention in Patients of CHD. *Glob J Health Sci* 2015;7:6-16.doi:10.5539/gjhs.v7n7p6

40. Salas-Salvadó J, Díaz-López A, Basora J, *et al.* Effect of a lifestyle intervention program with energy-restricted Mediterranean diet and exercise on weight loss and cardiovascular risk factors: One-year results of the PREDIMED-Plus trial. *Diabetes Care* 2019;42:777-788.doi:10.2337/dc18-0836

41. Gallagher R, Kirkness A, Armari E, *et al.* Weight management issues and strategies for people with high cardiovascular risk undertaking an Australian weight loss program: a focus group study. *Nurs Health Sci* 2012;14:18-24.doi:10.1111/j.1442-2018.2011.00651.x

42. Alsaleh E, Windle R, Blake H. Behavioural intervention to increase physical activity in adults with coronary heart disease in Jordan. *BMC Public Health* 2016;16:643.doi:10.1186/s12889-016-3313-5

43. Karyotaki E, Ebert DD, Donkin L, *et al.* Do guided internet-based interventions result in clinically relevant changes for patients with depression? An individual participant data meta-analysis. *Clin Psychol Rev* 2018;63:80-92.doi:10.1016/j.cpr.2018.06.007

44. Johansson P, Westas M, Andersson G, *et al.* An Internet-Based Cognitive Behavioral Therapy Program Adapted to Patients With Cardiovascular Disease and Depression: Randomized Controlled Trial. *JMIR Ment Health* 2019;6:e14648.doi:10.2196/14648

45. Safford MM, Reshetnyak E, Sterling MR, *et al.* Number of Social Determinants of Health and Fatal and Nonfatal Incident Coronary Heart Disease in the REGARDS Study. *Circulation* 2021;143:244-253.doi:10.1161/circulationaha.120.048026

46. Su JJ, Yu DS. Effects of a nurse-led eHealth cardiac rehabilitation programme on health outcomes of patients with coronary heart disease: A randomised controlled trial. *Int J Nurs Stud* 2021;122:104040.doi:10.1016/j.ijnurstu.2021.104040

For peer review only - http://bmjopen.bmj.com/site/about/guidelines.xhtml

47. Poli A, Kelfve S, Klompstra L*, et al.* Prediction of (Non)Participation of Older People in Digital Health Research: Exergame Intervention Study. *J Med Internet Res* 2020;22:e17884.doi:10.2196/17884

48. Arvidsson S, Bergman S, Arvidsson B*, et al.* Effects of a self-care promoting problem-based learning programme in people with rheumatic diseases: a randomized controlled study. *J Adv Nurs* 2013;69:1500-1514.doi:10.1111/jan.12008

49. Gucciardi E, DeMelo M, Offenheim A*, et al.* Patient factors associated with attrition from a self-management education programme. *J Eval Clin Pract* 2007;13:913-919.doi:10.1111/j.1365-2753.2006.00773.x

50. Nielsen SF, Nordestgaard BG. Negative statin-related news stories decrease statin persistence and increase myocardial infarction and cardiovascular mortality: a nationwide prospective cohort study. *Eur Heart J* 2016;37:908-916.doi:10.1093/eurheartj/ehv641

51. Hjelmfors L, Abrandt Dahlgren M, Kärner A*, et al.* Problem-based learning used in the context of cardiac rehabilitation: different scenes and different roles. *Australia*, 2014: 36, p.218-232. Taylor & Francis.

52. Köhler AK, Nilsson S, Jaarsma T*, et al.* Health beliefs about lifestyle habits differ between patients and spouses 1 year after a cardiac event - a qualitative analysis based on the Health Belief Model. *Scand J Caring Sci* 2017;31:332-341.doi:10.1111/scs.12351

53. Horder M, Andersen S. Patient education–a health technology assessment. *Copenhagen: Danish Centre for Health Technology Assessment NBoH* 2009

For peer review only - http://bmjopen.bmj.com/site/about/guidelines.xhtml

Figure Legend:

Figure 1. Flowchart on study participation.

Figure 2. Distribution of outcome variables in PBL and control groups at baseline 1, 3 and 5 year follow-up.

Supplemental Material 1

Supplemental Material 2

For peer review only

For peer review only - http://bmjopen.bmj.com/site/about/guidelines.xhtml

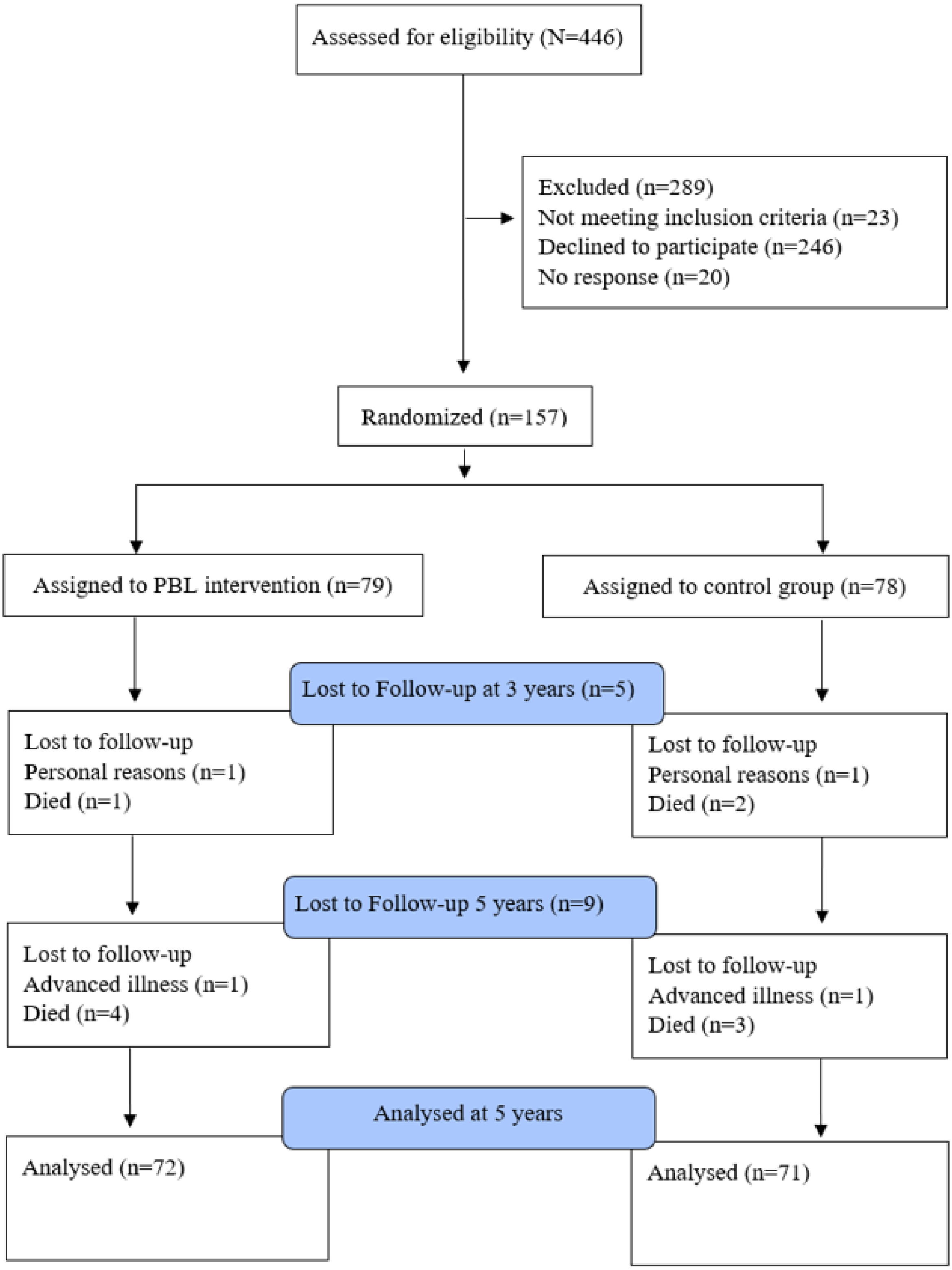

Figure 1. Flowchart on study participation

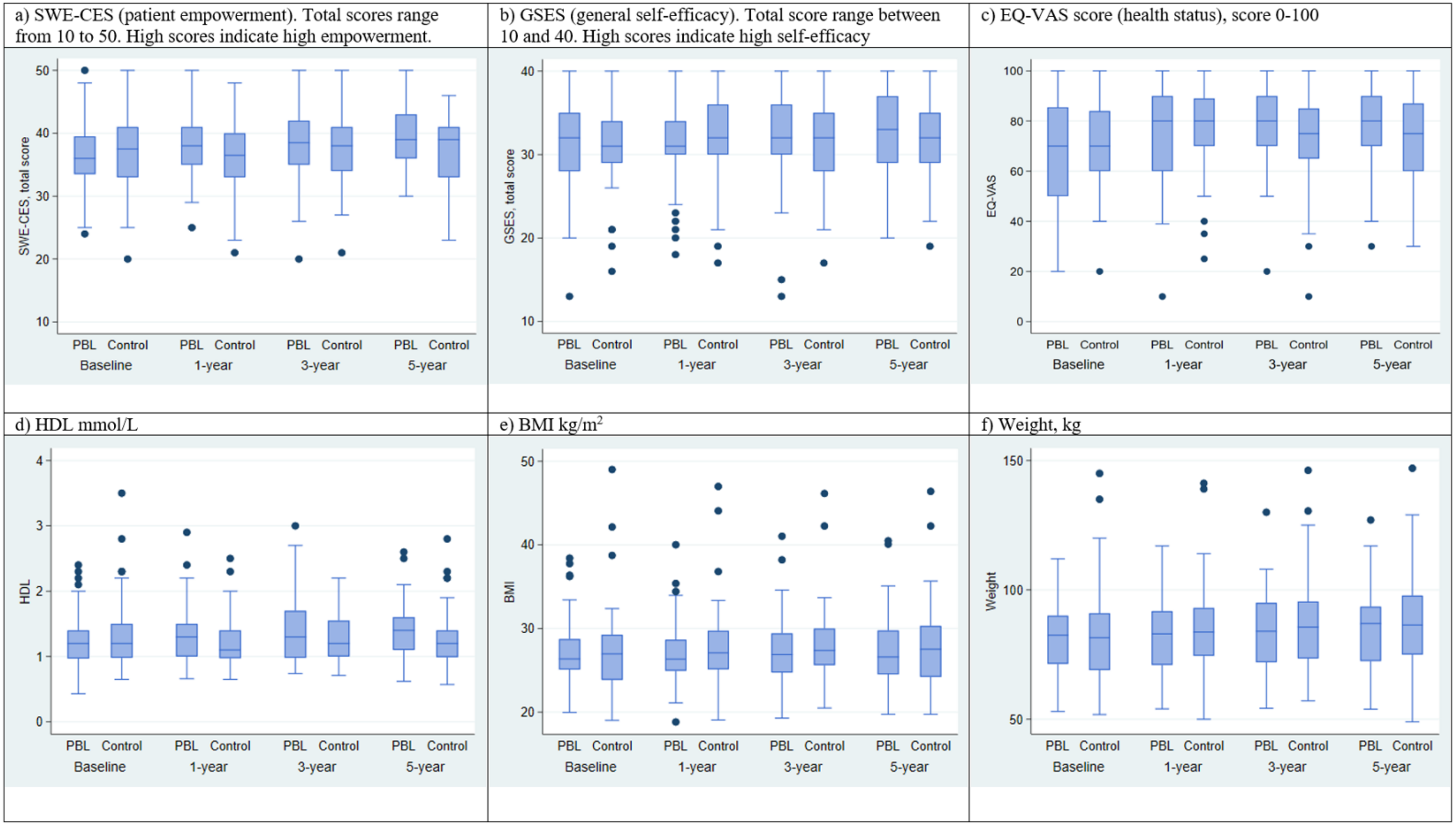

Figure 2. Distribution of outcome variables in PBL and control groups at baseline 1, 3 and 5 year follow-up.

Supplemental Material 1

COR-PRIM study
Department of social – and welfare studies
Linköping University, Campus Norrköping
2013. Anita Kärner phone: 011–363506

Responding letter regarding participation in the COR-PRIM study

From: Patients name…………………………………………………… (Written before sending out)

For about a week ago you received a written patient information about the COR-PRIM study, a test of a new treatment with pedagogical elements in primary care. Thereafter you were contacted by Anita Kärner, university lecturer and responsible for the study, and responded orally yes/wanted to consider more about the enquiry of participating in the study.
For you to be able to participate a written answer is needed as well. Put an X in a suitable box below. We want to emphasize that participation is totally voluntarily, and you can without motivation abstain from answering this request.

☐ I want to participate

☐ I do not want to participate. Reason (voluntarily information).

☐ I wish to be contacted again for more information before I decide. This box can also be filled in for you who want to participate. You can write question on the backside of this response letter

Date:…………………………………………………..

Signature:………………………………………………………………………………………

For you who want to be contacted again. Write telephone number and times when you can be contacted again on the backside of this letter.

Anita Kärner
University lecturer
Department of social – and welfare studies
Campus Norrkoping
Linköping University
Anita.Karner@liu.se
Phone: 011–363506

Staffan Nilsson
General practitioner
Primary Care Vikbolandet
County council Östergötland
Staffan.Nilsson@lio.se
Phone: 010–1030000

For peer review only

Supplemental Material 2 *BMJ open*

Title of the article:
Does problem-based learning improve patient empowerment and cardiac risk factors in patients with coronary heart disease in a Swedish primary care setting? A long-term prospective, randomized, parallel single randomized trial (COR-PRIM).

Authors: Christina Andreae, Pia Tingström, Staffan Nilsson, Tiny Jaarsma, Nadine Karlsson and Anita Kärner Köhler.

Supplement 2. Description of the PBL intervention

The PBL intervention consisted of group learning sessions in primary care led by district nurses who were advanced trained in PBL for two days by the research group. Two of the authors (AKK and PT) supported and tutored the district nurses every month in their development as PBL tutors. Tutoring groups consisted of 6-9 patients and participated in 13 learning sessions (2 hours) over one year. The sessions were every week for the first month, the two next months included two sessions/month, and the next five sessions were at 16, 20, 26, 39, 52 weeks after the start.

A problem-solving process (a PBL-model) supported the patient's formulation of mutual questions and learning goals and individual self-care goals, which they wrote and reflected on in an individual study guide. Triggers e.g., texts including real life scenarios or images were used to start the learning process about CHD, symptoms such as: chest pain, shortness of breath, swollen legs and signs of high blood pressure, cholesterol levels, lifestyle changes, in diet, physical activity, medicines, mental outcomes e.g., depressive mood, anxiety and fears, job life and stress, sex life. Importantly, the patients chose in which order the different scenarios were chosen to work with. This was a way to empower them and make the learning relevant to their situation. The district nurses supported and challenged the patients to select evidence-based literature. When the patients could not solve the questions, resource persons e.g., a general practitioner, physiotherapist were invited. These meetings were in between the group meetings and interested relatives were welcome and did attend.

Patients randomized to the control group received with the same interval as the PBL group, home-sent evidence-based patient information about self-care reflecting a cognitive intervention but did not participate in critical or reflective learning. Group effects were assessed after 11 sessions over one year.

For peer review only

CONSORT 2010 checklist of information to include when reporting a randomised trial*

Section/Topic	Item No	Checklist item	Reported on page No
**Title and abstract**			
	1a	Identification as a randomised trial in the title	1
	1b	Structured summary of trial design, methods, results, and conclusions (for specific guidance see CONSORT for abstracts)	2,3
**Introduction**			
Background and objectives	2a	Scientific background and explanation of rationale	3-5
	2b	Specific objectives or hypotheses	5
**Methods**			
Trial design	3a	Description of trial design (such as parallel, factorial) including allocation ratio	6-7
	3b	Important changes to methods after trial commencement (such as eligibility criteria), with reasons	NA
Participants	4a	Eligibility criteria for participants	6
	4b	Settings and locations where the data were collected	6
Interventions	5	The interventions for each group with sufficient details to allow replication, including how and when they were actually administered	7+ Supplement material +Figure 1
Outcomes	6a	Completely defined pre-specified primary and secondary outcome measures, including how and when they were assessed	7-8 and page 6
	6b	Any changes to trial outcomes after the trial commenced, with reasons	NA
Sample size	7a	How sample size was determined	7
	7b	When applicable, explanation of any interim analyses and stopping guidelines	NA
Randomisation:			
Sequence generation	8a	Method used to generate the random allocation sequence	6-7
	8b	Type of randomisation; details of any restriction (such as blocking and block size)	7
Allocation concealment mechanism	9	Mechanism used to implement the random allocation sequence (such as sequentially numbered containers), describing any steps taken to conceal the sequence until interventions were assigned	7
Implementation	10	Who generated the random allocation sequence, who enrolled participants, and who assigned participants to	7

For peer review only

		interventions	
Blinding	11a	If done, who was blinded after assignment to interventions (for example, participants, care providers, those assessing outcomes) and how	7
	11b	If relevant, description of the similarity of interventions	NA
Statistical methods	12a	Statistical methods used to compare groups for primary and secondary outcomes	9,10
	12b	Methods for additional analyses, such as subgroup analyses and adjusted analyses	10
**Results**			
Participant flow (a diagram is strongly recommended)	13a	For each group, the numbers of participants who were randomly assigned, received intended treatment, and were analysed for the primary outcome	Figure 1
	13b	For each group, losses and exclusions after randomisation, together with reasons	Figure 1
Recruitment	14a	Dates defining the periods of recruitment and follow-up	6
	14b	Why the trial ended or was stopped	NA
Baseline data	15	A table showing baseline demographic and clinical characteristics for each group	Table 1
Numbers analysed	16	For each group, number of participants (denominator) included in each analysis and whether the analysis was by original assigned groups	Table 2
Outcomes and estimation	17a	For each primary and secondary outcome, results for each group, and the estimated effect size and its precision (such as 95% confidence interval)	NA
	17b	For binary outcomes, presentation of both absolute and relative effect sizes is recommended	NA
Ancillary analyses	18	Results of any other analyses performed, including subgroup analyses and adjusted analyses, distinguishing pre-specified from exploratory	16-18 + Table 2
Harms	19	All important harms or unintended effects in each group (for specific guidance see CONSORT for harms)	NA
**Discussion**			
Limitations	20	Trial limitations, addressing sources of potential bias, imprecision, and, if relevant, multiplicity of analyses	20-22
Generalisability	21	Generalisability (external validity, applicability) of the trial findings	22
Interpretation	22	Interpretation consistent with results, balancing benefits and harms, and considering other relevant evidence	20-22
**Other information**			
Registration	23	Registration number and name of trial registry	6
Protocol	24	Where the full trial protocol can be accessed, if available	Ref 17
Funding	25	Sources of funding and other support (such as supply of drugs), role of funders	23

For peer review only - http://bmjopen.bmj.com/site/about/guidelines.xhtml

*We strongly recommend reading this statement in conjunction with the CONSORT 2010 Explanation and Elaboration for important clarifications on all the items. If relevant, we also recommend reading CONSORT extensions for cluster randomised trials, non-inferiority and equivalence trials, non-pharmacological treatments, herbal interventions, and pragmatic trials. Additional extensions are forthcoming: for those and for up to date references relevant to this checklist, see www.consort-statement.org.

For peer review only
